# Effects of a yoga-based stress reduction intervention on stress, psychological outcomes and cardiometabolic biomarkers in cancer caregivers: A randomized controlled trial

**DOI:** 10.1371/journal.pone.0277009

**Published:** 2022-11-10

**Authors:** Lena J. Lee, Robert Shamburek, Hyojin Son, Gwenyth R. Wallen, Robert Cox, Sharon Flynn, Li Yang, Margaret Bevans, Leslie Wehrlen, Alyson Ross

**Affiliations:** 1 Translational Biobehavioral and Health Disparities Branch, National Institutes of Health (NIH) Clinical Center, Bethesda, Maryland, United States of America; 2 National Heart, Lung, and Blood Institute, NIH, Bethesda, Maryland, United States of America; 3 Office of Research Support & Compliance, NIH Clinical Center, Bethesda, Maryland, United States of America; Fondazione Toscana Gabriele Monasterio, ITALY

## Abstract

Caregiving stress is a risk factor for cardiometabolic disease. Therefore, integrating cardiometabolic biomarkers into caregiving research provides a more comprehensive assessment of an individual’s health and response to an intervention. The objective of this study was to examine the effects of a yoga-based stress reduction intervention on stress, psychological outcomes, and cardiometabolic biomarkers in cancer caregivers. This prospective randomized controlled trial enrolled family caregivers of adult patients who underwent an allogeneic HSCT at the National Institutes of Health (NIH) Clinical Center. All subjects received usual care education. Participants in the intervention group received an Iyengar yoga intervention self-administered over six weeks using an audio recording file. The primary outcome was perceived stress (measured using the NIH toolbox Perceived Stress). The secondary outcomes were psychological factors (depression and anxiety measured using PROMIS^®^ Depression and Anxiety), and cardiometabolic biomarkers measured by nuclear magnetic resonance spectroscopy. A total of 50 family caregivers (mean [SD] age, 44.9 [15.2] years; 42 [84.0%] women) were randomized, 25 to the intervention group and 25 to the control group. No group differences were noted in stress, depression, and anxiety. Significant interaction effects between group and time were found in large TRL-P (*F*(1,43) = 10.16, *p* = 0.003) and LP-IR (*F*(1,42) = 4.28, *p* = 0.045). Post-hoc analyses revealed that the levels of large TRL-P (mean difference = 1.68, CI = [0.86, 2.51], *p<* .001) and LP-IR (mean difference = 5.67, CI = [1.15, 10.18], *p =* 0.015) significantly increased over time in the control group but while remained stable in the intervention group (mean difference = -0.15, CI = [-0.96, 0.66], *p* = 0.718; mean difference = -0.81, CI = [-5.22, 3.61], *p* = 0.714, respectively). Even when perceptions of psychological distress remain unchanged, incorporating gentle yoga poses and breathing exercises may reduce the risk of cardiometabolic disease in caregivers by inhibiting the development of insulin resistance. Standard lipids of cardiometabolic risk do not appear to be robust enough to detect short-term early changes of cardiometabolic risk in caregivers.

**Trial registration**: ClinicalTrials.gov Identifier: NCT02257853.

## Introduction

Caring for a family member with cancer, the fourth main reason for caregiving in the U.S., is intense and challenging [1, 2]. Among different types of cancer treatment, hematopoietic stem cell transplantation (HSCT), especially allogeneic, is a risky procedure that may cause serious early and late effects including graft versus host disease and cytopenia [3]. Caring for a HSCT recipient is accepted as burdensome, considering that the recipient needs extensive support [4, 5]. HSCT caregivers often experience high levels of stress and symptoms such as depression and anxiety due to their caregiving burden [6, 7]. Unmanaged stress and symptoms may increase the risk of developing cardiometabolic disease such as type 2 diabetes (T2D) and coronary artery disease in these caregivers [8–11].

Many of the intervention studies to relieve stress and symptoms in family caregivers of people with cancer took mindfulness approaches, often including yoga, breathing exercise, and meditation as components of the intervention programs [12–15]. These studies have shown that mindfulness-based interventions are effective in improving multiple symptoms (e.g., anxiety, depression, fatigue, sleep disturbance) and mental health in cancer caregivers [12–15]. However, such intervention studies in HSCT caregivers have been lacking. Furthermore, those studies have relied heavily on self-reported outcomes to evaluate the effects of the interventions, while physiological responses have been rarely included as outcomes. Specifically, there is a growing body of evidence that caregiving stress is a risk factor for the development of cardiometabolic disease [8, 16]; however, intervention studies focusing on assessing biomarkers of cardiometabolic function in the caregiver population are lacking.

Recent evidence has suggested incorporating a serum lipoprotein particle profile assessed via nuclear magnetic resonance (NMR) spectroscopy in order to precisely identify lipid-associated cardiometabolic risk because standard lipids can vary greatly in cholesterol content (i.e., particle concentration and size) among individuals [17, 18]. This approach measures particle concentrations and sizes of all lipoprotein classes (triglyceride-rich lipoprotein particle [TRL-P], low-density lipoprotein particle [LDL-P], and high-density lipoprotein particle [HDL-P]) and particle concentrations of lipoprotein subclasses (e.g., large, medium, small) [17]. Evidence has been accumulating, suggesting that NMR lipoprotein particle concentration and size may be sensitive enough to detect early signs of cardiometabolic risk [17–20]. However, to date, only two studies have demonstrated the superiority of NMR lipoprotein particle profile analysis in detecting early changes in cardiometabolic health in cancer caregivers [10, 21]. Furthermore, no published studies have examined whether yoga designed to decrease caregivers’ stress results in changes in cardiometabolic health measured by NMR lipoprotein particle profiles. Expanding the scientific research of lipoprotein particle profiles in caregivers will contribute to understanding the impact of caregiving stress on the caregivers’ cardiometabolic health and enhance our ability to evaluate interventions.

To reduce perceived stress, stress-related symptoms, and cardiometabolic risk in HSCT caregivers, we developed a six-week yoga-based stress reduction intervention. We assessed the effects of the intervention on the following outcomes: perceived stress and psychological outcomes (depression, anxiety) using self-reported measures; and cardiometabolic risk using NMR lipoprotein particle profile analysis.

## Methods

### Study design and participants

A prospective randomized controlled trial (RCT) design was used to examine the effectiveness of a six-week yoga-based stress reduction intervention in family caregivers of adult patients who underwent an allogeneic HSCT at the National Institutes of Health (NIH) Clinical Center (NCT#02257853). This study followed the Consolidated Standards of Reporting Trials (CONSORT) reporting guideline for RCTs. Participants were recruited between January 2015 and February 2019. Caregivers were eligible to participate in the study if they (1) were at least 18 years old; (2) were an active caregiver for a patient undergoing the 1^st^ allogeneic HSCT; (3) were able to read and speak English; (4) were able to lift arms over head without pain; and (5) were able to sit and stand from a seated position unassisted. This study was approved by the National Heart, Lung, and Blood Institute intramural Institutional Review Board. Written informed consent was obtained from all participants prior to initiating any study procedures.

### Intervention details

Based on prior research suggesting that cancer caregivers are less likely to participate in health-promoting behaviors that require them to leave the presence of the care recipient [22–24], we sought to develop a brief yoga intervention that could be performed anywhere without yoga props or special equipment. The six-week length of the intervention, as well as the 20-minute time of the audio file, was selected based upon the length of similar interventions in cancer caregiver populations, with interventions ranging between six to eight weeks [12–15]. We worked with a certified Advanced Iyengar yoga instructor with experience in the therapeutic use of yoga as well as extensive experience in developing audio yoga classes to develop the intervention. Iyengar yoga utilizes highly standardized teaching methods, and is the yoga style most often used in RCTs [25]. The intervention consisted of approximately ten minutes of standing poses at the wall (urdhva hastasana, adho mukha svanasana) and very gentle seated backbends and twists, followed by ten minutes of seated ujjayi pranayama (breath awareness) and seated savasana (relaxation pose). To ensure that participants could safely complete the intervention, the intervention participants were instructed individually on performance of every pose, and they were required to return demonstrate proper and safe performance of the poses. Intervention participants were asked to contact study staff immediately should they experience any discomfort or injury.

### Allocation, randomization, and details of group assignment

After study enrollment, the study statistician randomized participants to either the control or the intervention group using a permuted block randomization with allocation ratio of 1:1. All participants received usual care education, which includes transplant specific information for the recipient and the caregiver, as well as written and online caregiver resources. All participants met 1:1 at the baseline clinic visit with a study investigator to review the usual care education materials. In addition, participants in the intervention group received the audio file intervention, a pamphlet containing pictures of the proper performance of the poses, and a practice diary to record daily practice. The intervention participants were asked to practice daily and to record their practice in the practice diary.

### Outcome measures

#### Primary outcome

*Perceived stress*. The NIH toolbox was used to collect measures of perceived stress (10-item fixed form), using a 5-point Likert scale ranging from 1 to 5, with higher scores indicative of higher levels of perceived stress. Scores 1 standard deviation (SD) or more below the mean (T-score ≤ 40) indicate low levels of perceived stress and scores 1 SD or more above the mean (T-score ≥ 60) indicate high levels of perceived stress [26, 27].

#### Secondary outcomes

*Depression and anxiety*. The Patient-Reported Outcomes Measurement Information System (PROMIS^®^) is a reliable and highly validated system of self-reported health outcome measures [28]. In this analysis, PROMIS^®^ measures of depression and anxiety were administered using Computer Adaptive Testing (CAT) format. CAT uses validated algorithms to adapt a test based on the participant’s preceding responses. PROMIS^®^ measures generate a raw score from which T-scores are calculated, which are standardized scores that are normed to the general population with a mean of 50 and a SD of 10.

*Cardiometabolic biomarkers*. A lipoprotein particle profile was quantified from blood serum samples using a further-optimized deconvolution algorithm, called the fourth-generation lipoprotein profile algorithm, NMR spectroscopy [17]. The lipid biomarkers were calculated from the amplitudes of their spectroscopically unique lipid methyl group NMR signals. Particle concentration, concentration of subclasses by size (e.g., large, medium, small), and particle size were measured for all lipoprotein classes (TRL-Ps, LDL-Ps, HDL-Ps). Glycosylated acute phase proteins (GlycA), a novel inflammatory biomarker, reflects both increased glycan complexity and circulating acute-phase protein levels during local and systematic inflammation [17]. Lipoprotein insulin resistance (LP-IR), a novel composite metabolomic biomarker, captures the multidimensional effects of insulin resistance (IR) on the lipoprotein metabolic chain. Six lipoprotein parameters showing the strongest association with IR and prediabetes, including large TRL-P, TRL particle size (TRL-Z), small LDL-P, large HDL-P, LDL particle size (LDL-Z), and HDL particle size (HDL-Z), were used to derive LP-IR scores. LP-IR scores range from 0 (*Most insulin sensitive*) to 100 (*Most insulin resistant*) [17].

### Statistical analysis

Power analysis was conducted based on the primary end point of perceived stress. We used a two-side hypothesis that the perceived stress level would differ between the intervention group and the control group. Based on a Cohen’s d effect size of 0.6 and correlation of 0.8 between two time points from our previous study [29], with 80% power and type I error of 0.05, to detect a time-averaged difference between the intervention group and the control group, we estimated that we need 39 participants in each group [30]. However, after four years of recruiting and changes to the HSCT transplant program, we experienced challenges of recruitment and closed the study to further recruitment. Descriptive statistics (mean and SD for normally distributed continuous data, median and interquartile range for ordinal and non-normal data, frequencies and percentages for categorical data) were used to describe the demographic characteristics, perceived stress, psychological outcomes, and NMR-measured cardiometabolic biomarkers at the baseline and after six weeks of the intervention. Linear mixed models with time, group, and time by group interaction were used to analyze the effects of the intervention on the outcome measures over time. All variables at the baseline were tested if they were significantly different between the intervention and control groups. All data analyses were conducted using IBM SPSS statistics software, version 26.

## Results

**Fig 1:**
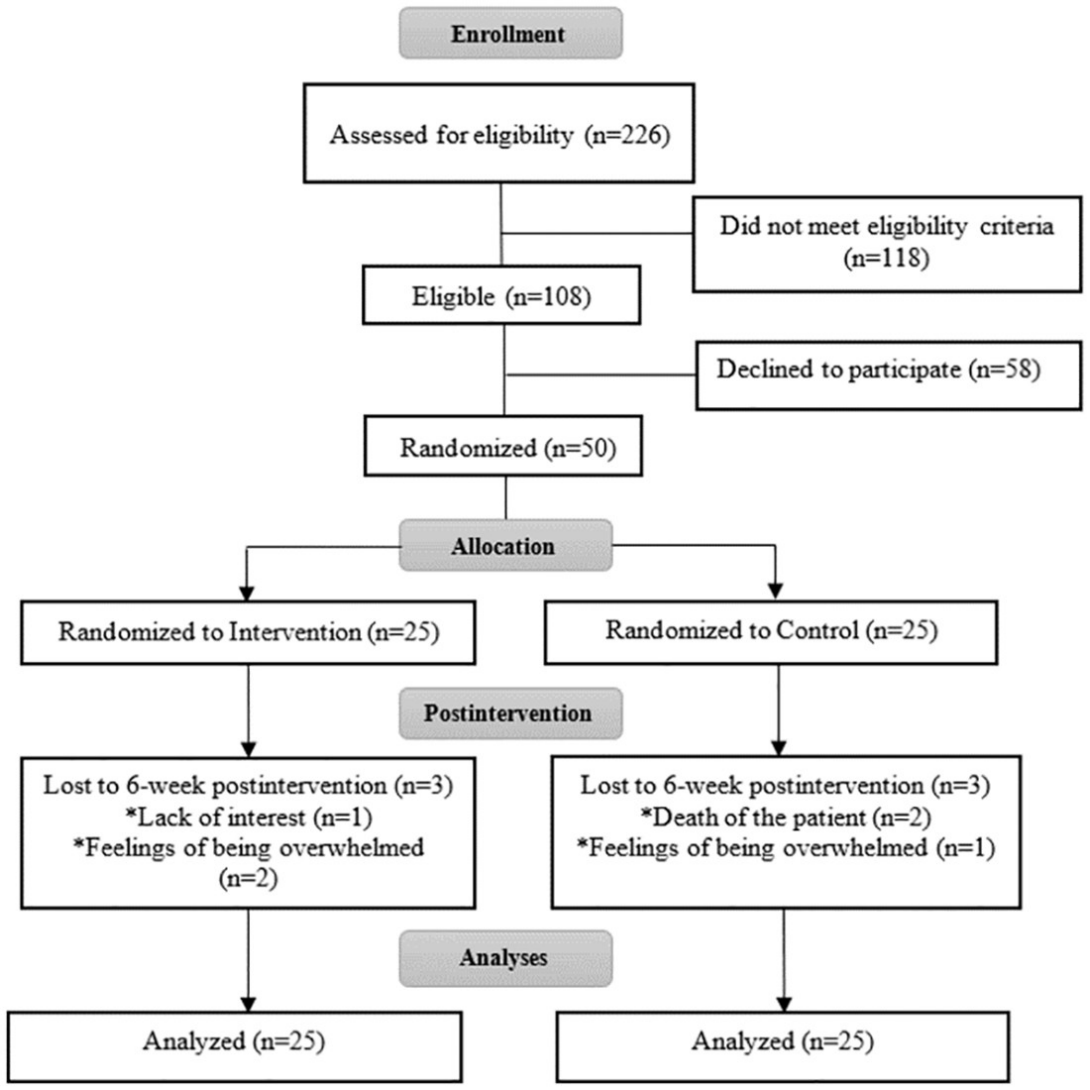
Consolidate Standards of Reporting Trials (CONSORT) flow diagram.

Of the 226 caregivers screened for the study, 108 were eligible and 50 agreed to participate, were enrolled, and then were randomly assigned to either the intervention group (n = 25) or the control group (n = 25) (Fig 1). Six of the 50 participants dropped out before completing the study because of lack of interest (n = 1), feelings of being overwhelmed (n = 3), and death of the patient (n = 2).

The baseline caregiver and patient characteristics of the two groups are detailed in Table 1. The groups did not differ on any of the baseline characteristics. The intervention group reported practicing the yoga intervention for 34.71 days (SD = 8.12), with a range of 16 to 42 days, and 652.95 minutes (SD = 159.08), with a range of 320 to 840 minutes. Throughout the six weeks of the intervention, the average weekly completion rates of those who completed the 20-minute intervention in its entirety ranged between 63% and 75%. No adverse effects were reported by the participants during the duration of the study. Cardiometabolic biomarkers are displayed in Table 2. The intervention group reported higher levels of large HDL-P than the control group at the baseline (*p =* 0.034). Otherwise, there were no significant differences between the two groups in cardiometabolic biomarkers.

**Table 1:** General characteristics at baseline (N = 50).

Variables	N (%) Mean (SD), range		
	Total (n = 50)	Intervention group (n = 25)	Control group (n = 25)
**Caregiver characteristics**			
Age (years)	44.94 (15.21), 18–74	47.32 (15.31), 18–74	42.56 (15.04), 18–66
Sex			
Female	42 (84.0)	22 (88.0)	20 (80.0)
Race/ethnicity			
White/Non-Hispanic	25 (50.0)	13 (52.0)	12 (48.0)
Non-White/Non-Hispanic	15 (30.0)	9 (36.0)	6 (24.0)
Hispanic	10 (20.0)	3 (12.0)	7 (28.0)
Marital status			
Married/cohabiting	39 (78.0)	21 (84.0)	18 (72.0)
Not married^a^	11 (22.0)	4 (16.0)	7 (28.0)
Annual household income			
< $50,000	20 (47.0)	10 (47.6)	10 (45.5)
$50,000-$89,000	7 (16.0)	4 (19.0)	3 (13.6)
> $89,000	16 (37.0)	7 (33.4)	9 (40.9)
Employment status			
Full-time	18 (36.0)	8 (32.0)	10 (40.0)
Part-time	12 (24.0)	6 (24.0)	6 (24.0)
Not working^b^	20 (40.0)	11 (44.0)	9 (36.0)
Changes in caregiver employment			
Yes	40 (80.0)	20 (80.0)	20 (80.0)
No	10 (20.0)	5 (20.0)	5 (20.0)
BMI (kg/m^2^)	28.13 (6.15), 18.5–46.5	27.26 (5.81), 18.50–44.4	29.01 (6.46), 19.7–46.5
Waist circumference			
Male	100.38 (8.75), 88–111	100.67 (7.29), 92.5–106.5	100.20 (10.35), 88–111
Female	90.58 (14.30), 65–136	90.92 (15.84), 65–136	90.21 (12.79), 70–124
SBP (mg/dL)	113.97 (13.31), 88–144	113.53 (12.24), 94–144	114.41 (14.53), 88–143
DBP (mg/dL)	67.37 (9.50), 49–91	66.84 (8.88), 49–84	67.90 (10.25), 51–91
Chronic health problems			
Yes	36 (72.0)	17 (68.0)	19 (76.0)
No	14 (28.0)	8 (32.0)	6 (24.0)
Relationships with patient			
Spouse	25 (50.0)	12 (48.0)	13 (52.0)
Non-spouse	25 (50.0)	13 (52.0)	12 (48.0)
Caregiving days/week	6.76 (1.03), 2–7	7.00 (0.00), 0–7	6.52 (1.42), 2–7
Caregiving hours/day	14.30 (7.89), 1.50–24.0	14.44 (8.13), 1.5–24	14.16 (7.82), 2–24
Perceived stress^c^	54.83 (10.62), 30.77–76.13	53.49 (11.44), 34.78–76.13	56.17 (9.78), 30.78–74.78
Depression^d^	51.04 (7.78), 34.17–71.37	49.23 (7.76), 34.17–71.37	52.85 (7.51), 41.73–69.10
Anxiety^d^	60.46 (7.01), 44.55–75.54	60.70 (6.94), 44.55–73.44	60.22 (7.21), 46.69–75.54
**Patient characteristics**			
Age (years)	36.68 (14.03), 18–66	36.20 (13.48), 18–66	37.16 (14.83), 20–65
Patient sex			
Male	33 (66.0)	16 (64.0)	17 (68.0)
Primary disease^e^			
Hematological malignancy	26 (52.0)	10 (40.0)	16 (64.0)
Non-hematological malignancy	24 (48.0)	15 (60.0)	9 (36.0)
Type of transplant			
RIC	32 (64.0)	18 (72.0)	14 (56.0)
Myeloablative	18 (36.0)	7 (28.0)	11 (44.0)
Stem cell source			
Peripheral blood	39 (78.0)	19 (76.0)	20 (80.0)
Bone marrow	10 (20.0)	5 (20.0)	5 (20.0)
Cord	1 (2.0)	1 (4.0)	0 (0.0)
Disease risk category			
Low	39 (78.0)	22 (88.0)	17 (68.0)
Intermediate	5 (10.0)	1 (4.0)	4 (16.0)
High	6 (12.0)	2 (8.0)	4 (16.0)

Note. Numbers may not sum to total due to missing data.

BMI, body mass index; DBP, diastolic blood pressure; SBP, systolic blood pressure; RIC, reduced intensity conditioning.

^a^Not married = never married, divorced, separated, widowed,

^b^Not working = student, retired, disability, unemployed,

^c^Assessed using NIH toolbox,

^d^Assessed using Patient-Reported Outcome Measurement Information System^®^ (PROMIS^®^),

^e^Primary disease, hematological malignancy = chronic myelogenous leukemia, acute lymphocytic leukemia, acute myelogenous leukemia, and chronic lymphocytic leukemia, Hodgkin’s and non-Hodgkin’s lymphoma; non-hematological malignancy = aplastic anemia, sickle cell disease, inherited bone marrow failure disorders, primary immunodeficiency disease.

**Table 2:** Levels of cardiometabolic biomarkers in intervention and control groups at baseline.

Variables	Intervention group (n = 25)		Control group (n = 25)		Refence range value^a^	
	Mean (SD)	Range	Mean (SD)	Range	Mean (SD)	Range
**Triglyceride-rich Particles (nmol/L)**						
Total TRL-P	115.61 (96.99)	15.7–484.5	112.85 (70.91)	27.4–273.5	125.2 (61.6)	42–239
Very Large TRL-P	0.14 (.09)	0–0.3	0.27 (0.49)	0–2.5	0.4 (1.0)	0–1.6
Large TRL-P	1.20 (2.35)	0–11.4	1.6 (2.79)	0–13.9	2.9 (6.5)	0–12.8
Medium TRL-P	17.96 (16.41)	0–57.6	18.28 (12.67)	0–40.6	17.9 (16.2)	0.3–48.4
Small TRL-P	45.68 (36.79)	0–147	29.62 (23.02)	4.6–99.7	56.6 (37.5)	7.3–124.4
Very Small TRL-P	50.63 (67.34)	0–281.20	63.08 (69.58)	0–230.8	47.5 (46.9)	0–142.3
**LDL Particles (nmol/L)**						
Total LDL-P	1557.6 (447.81)	1001–2626	14300 (369.39)	831–2398	1454.0 (393.0)	891–2150
Large LDL-P	239.72 (180.44)	0–631	158.12 (180.80)	0–727	309.0 (223.0)	17–748
Medium LDL-P	702.88 (381.85)	0–1528	597.96 (386.38)	0–1182	676.0 (405.0)	0–1377
Small LDL-P	615.12 (493.96)	71–2017	674 (594.13)	0–2134	469.0 (431.0)	13–1318
**HDL Particles (μmol/L)**						
Total HDL-P	19.7 (3.14)	14.1–24.7	19.39 (2.97)	14.6–24.8	24.0 (3.0)	19.2–29.3
Large HDL-P*	3.08 (1.99)	0.5–7.3	2.04 (1.44)	2.0–4.7	2.5 (1.9)	0.2–6.3
Medium HDL-P	3.97 (2.13)	1.2–9.2	3.54 (1.54)	1.0–6.6	7.7 (2.7)	3.7–12.6
Small HDL-P	12.65 (4.47)	2.4–19.8	13.80 (2.83)	7.7–19.8	13.8 (3.4)	8.1–19.6
**Mean Particles Sizes (nm)**						
TRL-Z	41.22 (6.19)	33.3–63.7	43.84 (7.00)	30.8–67.4	44.0 (8.4)	33.8–60.9
LDL-Z	20.91 (0.49)	19.8–21.6	20.76 (0.56)	19.7–21.7	21.0 (0.5)	20.1–21.7
HDL-Z	9.15 (0.44)	8.4–10	8.95 (0.36)	8.3–9.6	9.0 (0.4)	8.3–9.8
**Lipids and Apolipoproteins (mg/dL)**						
Total cholesterol	183.36 (37.04)	129–272	165.32 (29.80)	114–223	193.8 (36.5)	140–256
LDL-C	104.52 (29.28)	67–182	93.40 (23.18)	57–135	110.5 (30.7)	63–163
HDL-C	56.08 (13.69)	31–85	49.92 (12.35)	34–72	61.1 (14.4)	41–88
Triglycerides	109.00 (50.97)	51–244	107.88 (43.52)	51–237	119.3 (89.8)	43–276
Apolipoprotein A1	133.32 (21.71)	90–169	123.92 (21.91)	85–166	156.8 (27.8)	116–209
Apolipoprotein B	90.04 (27.18)	56–167	81.68 (20.93)	47–130	87.1 (23.6)	53–127
**Inflammatory Biomarker (μmol/L)**						
GlycA	374.92 (51.14)	283–510	367.16 (59.14)	258–478	402.4 (65.8)	307–524
**Composite Metabolomic Marker**						
LP-IR	27.76 (23.35)	1–94	38.28 (22.27)	3–92	36.0 (24.5)	3–83

Note. GlycA, glycoprotein acetylation; HDL-C, high-density lipoprotein cholesterol; HDL-P, high-density lipoprotein particles; HDL-Z, high-density lipoprotein size; LDL-C, low-density lipoprotein cholesterol, LDL-P, low-density lipoprotein particles; LDL-Z, low-density lipoprotein size; LP-IR, lipoprotein insulin resistance index; TRL-P, triglyceride rich lipoprotein particles; TRL-Z, triglyceride rich lipoprotein size.

^a^Reference range values are from a representative sampling (n = 698) of the general population, comprised of apparently healthy men (n = 284) and women (n = 414) aged 18 to 84 years (mean 39 years).

*p < .05

### Perceived stress

Six percent of our sample reported low levels of perceived stress and 32% reported high levels of perceived stress. There were no significant differences found for group or time, or the interaction between group and time for perceived stress. A post-hoc power analysis for the primary outcome was performed. The actual effect size for time averaged perceived stress between groups was approximately 0.4; the correlation between time points was approximately 0.8. Thus, with the sample size of 50, the actual power for the primary outcome was 32%, not the desired 80%.

### Depression and anxiety

There were no significant differences found for group or time, or the interaction between group and time for depression and anxiety.

### Cardiometabolic biomarkers

**Fig 2:**
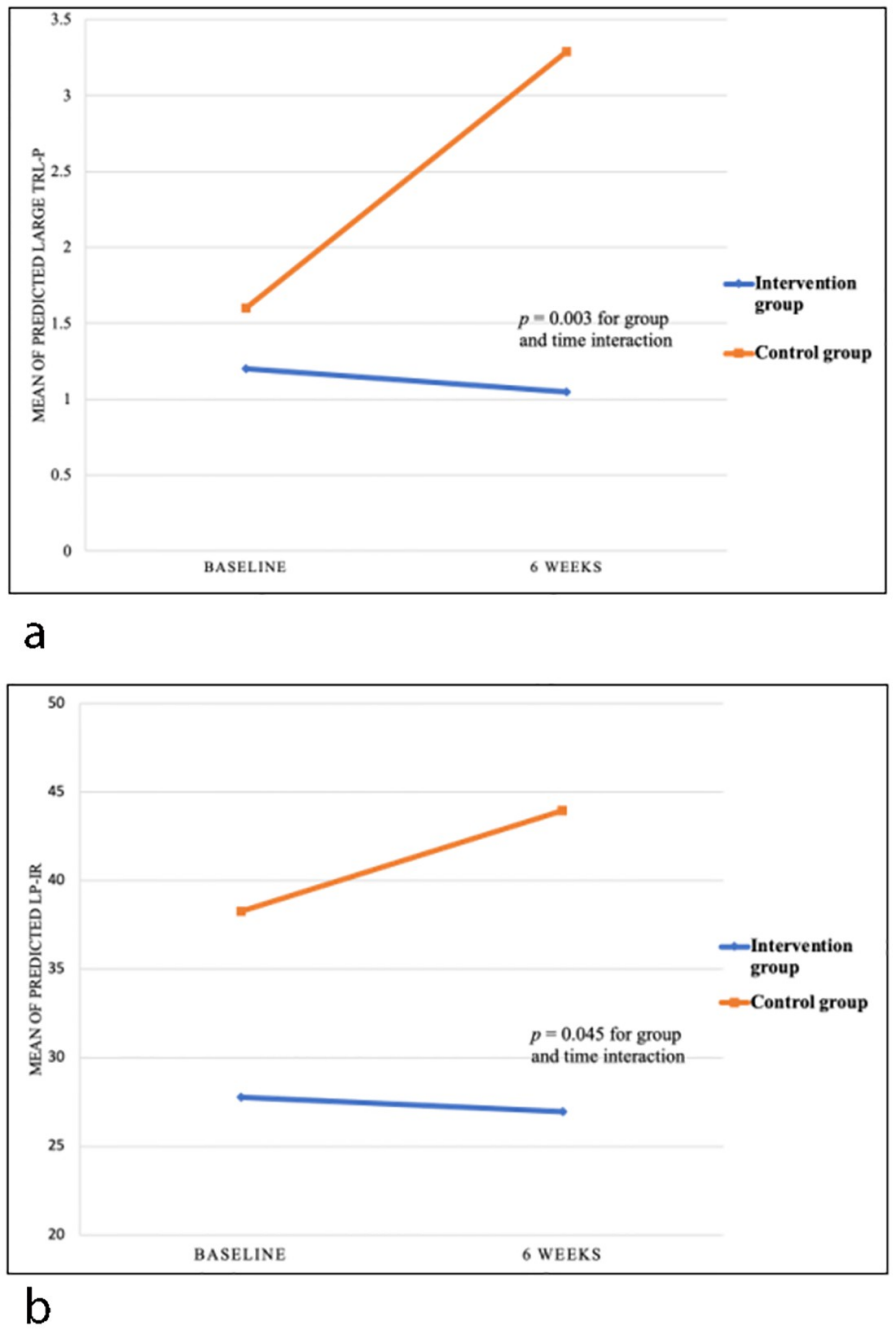
Mean cardiometabolic biomarkers levels over time for the intervention and control groups. a. Predicted large TRL-p overtime. Post-hoc analyses showed significant differences between baseline and 6 weeks for control group only (*p* < 0.001). b. Predicted LP-IR over time. Post-hoc analyses showed significant differences between baseline and 6 weeks for control group only (*p* = 0.015).

There were significant effects in two of cardiometabolic biomarkers, large TRL-P and LP-IR (Table 3). There was a significant interaction effect between group and time in large TRL-P (*F* (1,43) = 10.16, *p* = 0.003). The levels of large TRL-P significantly increased over time in the control group (mean difference [MD] = 1.68; confidence interval [CI] = [0.86, 2.51], *p <* .001) but did not significantly change in the intervention group (MD = -0.15; CI = [-0.96, 0.66], *p* = 0.718) (Fig 2a). A significant interaction effect between group and time was found in LP-IR (*F* (1,42) = 4.28, *p* = 0.045), with the trajectory of LP-IR increasing overtime in the control group (MD = 5.67; CI = [1.15, 10.18], *p =* 0.015) while a nonsignificant change in LP-IR for the intervention group (MD = -0.81; CI = [-5.22, 3.61], *p* = 0.714) (Fig 2b).

**Table 3:** Cardiometabolic biomarker outcomes that differed by group over time.

Outcome	Measurement	Intervention group	Control group	Mixed model analysis	
		Mean (SE)	Mean (SE)	Effect	*p* value
Large TRL-P	Baseline	1.20 (0.63)	1.60 (0.63)	**Time**	**0.010**
	6 weeks	1.05 (0.64)	3.29 (0.65)	Group	0.129
				**Group x time**	**0.003**
LP-IR	Baseline	27.76 (4.46)	38.28 (4.46)	Time	0.128
	6 weeks	26.95 (4.52)	43.95 (4.55)	**Group**	**0.030**
				**Group x time**	**0.045**

Note. Boldface indicates statistical significance (*p* < 0.05). HDL-P, high-density lipoprotein particles; TRL-P, triglyceride rich lipoprotein particles; LP-IR, lipoprotein insulin resistance index.

## Discussion

Our study is the first to examine the efficacy of a six-week yoga-based stress reduction intervention to improve perceived stress, psychological outcomes, and cardiometabolic health, as measured by novel NMR lipoprotein particle concentration and size, in HSCT caregivers. Multiple studies using yoga-based meditation and mindfulness interventions have shown beneficial effects on stress and psychological symptoms [12–14, 31–33]. However, no other study reported cardiometabolic outcomes in caregivers. In this study, there were no significant differences between the two groups in perceived stress, depression, and anxiety, nor were there within group changes in either of the two groups in these outcomes from the baseline to the trial’s end. Nevertheless, we found that the six-week yoga-based stress reduction intervention led to beneficial changes in the lipoprotein subclass profile, large TRL-P and LP-IR.

In our study, it is somewhat surprising that perceived stress and symptoms of depression and anxiety did not improve in the intervention group. Perceived stress is commonly examined in yoga intervention research and is believed to be positively influenced by engagement in yoga-based stress reduction [32, 33]. However, there are some conflicting reports regarding yoga-based intervention’s impact on psychological outcomes. Some studies showed beneficial results in psychological outcomes, such as depression and anxiety [12–14, 31–33], while other studies did not observe significant changes in these outcomes [34, 35]. One possible reason for the difference in findings between previous research and this study may be attributed to the relatively short-term intervention. Two systematic reviews assessing the effects of yoga-based stress reduction intervention in nonclinical populations, with the duration varying from four to 24 weeks, suggested that the intervention duration is an important factor of yoga effectiveness [36, 37]. The optimal duration of yoga-based stress reduction intervention is unclear, but studies with longer intervention periods appear to have better results [37]. Thus, further studies need to collect outcomes at multiple time points to ascertain the effects of longer-term intervention on psychological outcomes in caregivers. In addition, with 50 participants, this study was not sufficiently powered to detect differences in the patient reported outcome measures between groups. Future studies should be appropriately powered to assess the effects of perceived stress by recruiting more participants, enrolling participants at higher risk, and prolonging follow-up.

The most notable finding of this study was that the yoga-based stress reduction intervention appeared to be protective of negative changes in large TRL-P, which is specifically associated with IR. IR is a key risk factor of metabolic dysfunction in prediabetes and T2D, characterized by deterioration in tissue sensitivity to insulin and a compensatory increase in insulin secretion [38]. Impaired insulin sensitivity may contribute to occlusive vascular disease, which has been positively related to increased risk of coronary artery disease and ischemic stroke in the general population [39, 40]. It is well appreciated that worsening IR was associated with lipoprotein abnormalities featured by remarkably increased large VLDL-P, without consistent changes in medium or small VLDL-P [41, 42]. Large VLDL-P may be more critical in predicting atherogenic risk than medium or small VLDL-P since a greater proportion of large lighter VLDL is efficiently catabolized to small dense LDL particles, leading to an increase in serum triglycerides and cholesterol ester in the vascular intima [38, 43]. Using a novel NMR platform-derived algorithm that captures five different TRL subclasses, we found that HSCT caregivers in the control group showed a significant increase in circulating large TRL-P while levels in the intervention group remained unchanged. The findings suggested a potential mechanism by which caregiving stress may increase the risk of cardiometabolic disease. These findings are strengthened by our own past research finding that “double duty caregivers” who experienced the added stress of providing care to another individual in addition to the cancer patient were more likely to have higher levels of large TRL-P than were caregivers who only provided care to the cancer patient [10]. We also previously found that HSCT caregivers’ levels of large VLDL-P worsened over time, while the levels remained unchanged in non-caregivers, indicating very early signs of cardiometabolic disease in these caregivers [21]. Perhaps caregiving stress has a bigger impact on triglyceride-rich remnant lipoprotein, and it is through this pathway that caregivers are at increased cardiometabolic risk. One of the main determinants of circulating triglycerides is the level of lipoprotein lipase, which is well known to be affected by stress [44].

Another important finding in this study is that the yoga-based stress reduction intervention elicited notable beneficial changes in LP-IR, a novel composite metabolomic marker of IR and T2D risk. A growing body of clinical trials demonstrated that LP-IR scores were strongly associated with incidence T2D even independent of established risk factors including glucose or HBA1c levels [45–47]. Furthermore, LP-IR scores have been shown to predict future T2D in individuals at low risk for T2D based on their clinical profiles [45]. LP-IR scores offer a simple, reliable way to monitor a patient’s risk of T2D and the effectiveness of treatments that may prevent or delay the onset of T2D [45–47]. Previous studies evaluating the efficacy of modification interventions that incorporate diet, exercise, stress management or group support on cardiometabolic health have demonstrated the comparability of LP-IR with traditional markers for determining a patient’s IR state [48, 49]. However, no other study to date has assessed the effects of a yoga-based stress reduction intervention on LP-IR. In this study, a significant improvement in LP-IR was observed in caregivers in the intervention group compared to those in the education control group. As LP-IR was sensitive to change following the yoga-based stress reduction intervention, our findings provide evidence for the clinical utility of this novel composite metabolomic marker to assess alteration in diseases risk in caregivers. In addition, our findings demonstrated that the yoga-based stress reduction intervention was effective for improving IR defined by LP-IR scores, suggesting that yoga-based stress reduction may decrease the risk of cardiometabolic disease in caregivers by preventing or delaying the onset of T2D. The exact mechanisms underlying the effects of yoga-based stress reduction on diabetes risk profiles are not yet well understood, and further studies are warranted. This study has several limitations. First, with the small sample size, the study was underpowered to detect differences in the primary outcome between groups. These factors were accompanied by the relatively short data collection period, which needs to be considered when interpreting these findings. Further studies using larger samples are needed. Second, this study recruited only caregivers of individuals receiving HSCT at the NIH Clinical Center, a unique research hospital that provides care only to individuals enrolled in research protocols. Thus, the findings may not be generalizable to caregivers of patients receiving traditional care in general hospitals or clinics. In addition, while the study revealed a relatively high and consistent overall adherence to the intervention, monitoring the correct completion of the yoga poses is not possible when participants’ completion is self-paced outside of a controlled setting. Given the growing number of people using live streaming platforms, incorporating this technology (via the real-time display of video and synchronous communications) may provide monitoring of the intervention and permit instant feedback from participants during the practice improving intervention fidelity. Finally, since this study was designed to measure study outcomes only at the baseline and six weeks after the completion of the intervention, we did not measure study outcomes immediately before and after the 20-minute intervention. This would have allowed us to examine whether the intervention might have relieved levels of stress or psychological outcomes temporarily following the completion of the audio file intervention. This temporary respite may have been enough to protect the intervention participants from negative changes in cardiometabolic biomarkers. Assessment of measures across multiple time points (e.g., pre and post a single yoga session in addition to pre and post the entire study) and longer follow-up could further inform how stress or psychological outcomes change throughout the caregiving trajectory and which mechanisms may lead to changes in cardiometabolic health in HSCT caregivers.

## Conclusions

In this study, yoga-based stress reduction was effective for protecting cardiometabolic health in HSCT caregivers, possibly preventing or delaying progression toward T2D. We reported that detailed lipoprotein profiling might provide insight into the mechanisms underlying the protective influence of gentle yoga poses and breathing exercises in preventing negative changes in vascular atherogenicity and insulin sensitivity. Although additional research is needed to determine factors influencing long-term changes in IR, health care providers should consider the benefits of yoga-based stress reduction for improving insulin sensitivity and coronary artery disease risk when developing tailored strategies to maintain optimal cardiometabolic health of family caregivers.

## Supporting information

S1 Checklist CONSORT checklist. (DOC)

S1 File Study protocol. (DOCX)

## References

[1] National Alliance for Caregiving. Caregiving in the U.S. 2020. https://www.aarp.org/content/dam/aarp/ppi/2020/05/full-report-caregiving-in-the-united-states.

[2] National Alliance for Caregiving. Cancer Caregiving in the U.S.: An intense, episodic, and challenging care experience. https://www.caregiving.org/wp-content/uploads/2020/05/CancerCaregivingReport_FINAL_June-17-2016.pdf

[3] Arnaout K, Patel N, Jain M, El-Amm J, Amro F, Tabbara IA. Complications of allogeneic hematopoietic stem cell transplantation. Cancer Invest. 2014;32: 349–362. doi: 10.3109/07357907.2014.919301 24902046

[4] Applebaum AJ, Bevans M, Son T, Evans K, Hernandez M, Giralt S, et al. A scoping review of caregiver burden during allogeneic HSCT: lessons learned and future directions. Bone Marrow Transplant. 2016;51(11): 1416–1422. doi: 10.1038/bmt.2016.164 27295270 PMC5564416

[5] Xie L, Shen C, Shi Y, Li H. Caregiver burden among primary family caregivers of patients undergoing HSCT: a cross-sectional study from Suzhou, China. Cancer Nurs. 2021;44(6): E556–E566. 32976183 10.1097/NCC.0000000000000895

[6] Gupta V, Raj M, Hoodin F, Yahng L, Braun T, Choi SW. Electronic health record portal use by family caregivers of patients undergoing HSCT: United States national survey study. JMIR Cancer. 2021;7(1): e26509. 33687332 10.2196/26509 PMC8086639

[7] Ravyts SG, Sannes TS, Dzierzewski JM, Zhou ES, Brewer BW, Natvig C, et al. Check your sleep before you start: a secondary analysis of a stress management intervention for caregivers of stem cell transplant patients. Psychooncology. 2021;30(6): 936–945. doi: 10.1002/pon.5680 33749066 PMC8855839

[8] Ahn S, Esquivel JH, Davis EM, Logan JG, Chung ML. Cardiovascular disease incidence and risk in family caregivers of adults with chronic conditions: a systematic review. J Cardiovasc Nurs. 2022;37(3): E47–E60. doi: 10.1097/JCN.0000000000000816 33938535

[9] Kim Y, Carver CS, Shaffer KM, Gansler T, Cannady RS. Cancer caregiving predicts physical impairments: roles of earlier caregiving stress and being a spousal caregiver. Cancer. 2015;121(2): 302–310. doi: 10.1002/cncr.29040 25209592

[10] Lee LJ, Kim Y, Shamburek R, Ross A, Yang L, Bevans MF. Caregiving stress and burden associated with cardiometabolic risk in family caregivers of individuals with cancer. Stress. 2022;25(1): 258–266. doi: 10.1080/10253890.2022.2037548 35727023 PMC9380420

[11] Steel JL, Cheng H, Pathak R, Wang Y, Miceli J, Hecht CL, et al. Psychosocial and behavioral pathways of metabolic syndrome in cancer caregivers. Psychooncology. 2019;28(8): 1735–1742. doi: 10.1002/pon.5147 31206896 PMC6768062

[12] Johns SA, Beck-Coon K, Stutz PV, Talib TL, Chinh K, Cottingham AH, et al. Mindfulness training supports quality of life and advance care planning in adults with metastatic cancer and their caregivers: results of a pilot study. Am J Hosp Palliat Care. 2020;37(2): 88–99. doi: 10.1177/1049909119862254 31378080 PMC8112585

[13] Schellekens MPJ, van den Hurk DGM, Prins JB, Donders ART, Molema J, Dekhuijzen R, et al. Mindfulness-based stress reduction added to care as usual for lung cancer patients and/or their partners: a multicentre randomized controlled trial. Psychooncology. 2017;26(12): 2118–2126. doi: 10.1002/pon.4430 28337821

[14] Milbury K, Li J, Weathers S-P, Mallaiah S, Armstrong T, Li Y, et al. Pilot randomized, controlled trial of a dyadic yoga program for glioma patients undergoing radiotherapy and their family caregivers. Neurooncol Pract. 2019;6(4): 311–320. doi: 10.1093/nop/npy052 31386042 PMC6660820

[15] Vinci C, Pidala J, Lau P, Reblin M, Jim H. A mindfulness-based intervention for caregivers of allogeneic hematopoietic stem cell transplant patients: pilot results. Psychooncology. 2020;29(5): 934–937. doi: 10.1002/pon.5353 32043667

[16] Park J, Ross A, Klagholz SD, Bevans MF. The role of biomarkers in research on caregivers of cancer patients: a scoping review. Biol Res Nurs. 2018;20(3): 300–311. 29130313 10.1177/1099800417740970 PMC6346308

[17] Franke DDH, Connelly MA. Chapter 11—Nuclear magnetic resonance technology and clinical applications. In: Clarke W, Marzinke MA, editors. Contemporary practice in clinical chemistry. 4th ed. Academic Press; 2020. pp. 187–200.

[18] Otvos JD, Mora S, Shalaurova I, Greenland P, Mackey RH, Goff DC Jr. Clinical implications of discordance between low-density lipoprotein cholesterol and particle number. J Clin Lipidol. 2011;5(2): 105–113. doi: 10.1016/j.jacl.2011.02.001 21392724 PMC3070150

[19] Matyus SP, Braun PJ, Wolak-Dinsmore J, Jeyarajah EJ, Shalaurova I, Xu Y, et al. NMR measurement of LDL particle using the Vantera Clinical Analyzer. Clin Biochem. 2014;47(16–17): 203–210. 25079243 10.1016/j.clinbiochem.2014.07.015

[20] Urbina EM, McCoy CE, Gao Z, Khoury PR, Shah AS, Dolan LM, et al. Lipoprotein particle number and size predict vascular structure and function better than traditional lipids in adolescent and young adults. J Clin Lipidol, 2017;11(4): 1023–1031. 28826565 10.1016/j.jacl.2017.05.011 PMC5657457

[21] Ross A, Shamburek R, Wehrlen L, Klagholz SD, Yang L, Stoops E, et al. Cardiometabolic risk factors and health behaviors in family caregivers. PLoS One. 2017;12(5): e0176408. doi: 10.1371/journal.pone.0176408 28472106 PMC5417518

[22] Ross A, Lee LJ, Wehrlen L, Cox R, Yang L, Perez A, et al. Factors that influence health-promoting behaviors in cancer caregivers. Oncol Nurs Forum, 2020;47(6): 692–702. doi: 10.1188/20.ONF.692-702 33063787

[23] Mochari-Greenberger H, Mosca L. Caregiver burden and nonachievement of health lifestyle behaviors among family caregivers of cardiovascular disease patients. Am J Health Promot. 2012;27(2): 84–89. 23113777 10.4278/ajhp.110606-QUAN-241 PMC4041363

[24] Sisk RJ. Caregiver burden and health promotion. Int J Nurs Stud. 2000;37(1): 37–43. doi: 10.1016/s0020-7489(99)00053-x 10687808

[25] Cramer H, Ward L, Saper R, Fishbein D, Dobos G, Lauche R. The safety of yoga: a systematic review and meta-analysis of randomized controlled trials. Am J Epidemiol. 2015;182(4): 281–293. doi: 10.1093/aje/kwv071 26116216

[26] Cohen S, Janicki‐Deverts D. Who’s stressed? Distributions of psychological stress in the United States in probability samples from 1983, 2006, and 2009. J Appl Soc Psychol. 2012;42(6): 1320–1334.

[27] Gershon RC, Wagster MV, Hendrie HC, Fox NA, Cook KF, Nowinski CJ. NIH toolbox for assessment of neurological and behavioral function. Neurology. 2013;80(11 Suppl 3):S2–S6. doi: 10.1212/WNL.0b013e3182872e5f 23479538 PMC3662335

[28] Cella D, Riley W, Stone A, Rothrock N, Reeve B, Yount S, et al. The Patient-Reported Outcomes Measurement Information System (PROMIS) developed and tested its first wave of adult self-reported health outcome item banks: 2005–2008. J Clin Epidemiol. 2010;63(11): 1179–1194. doi: 10.1016/j.jclinepi.2010.04.011 20685078 PMC2965562

[29] Bevans MF, Ross A, Wehrlen L, Klagholz SD, Yang L, Childs R, et al. Documenting stress in caregivers of transplantation patients: initial evidence of HPA dysregulation. 2016;19(2): 175–184. 10.3109/10253890.2016.1146670 PMC4976925 26949170

[30] Fitzmaurice GM, Laird NM, Ware JH. Applied longitudinal analysis. 2^nd^ Edition. John Wiley & Sons; 2011. Chapter 20, Sample size and power for longitudinal studies; p. 581–609.

[31] Bernardi MLD, Amorim MHC, Salaroli LB, Zandonade E. Effects of Hatha Yoga on caregivers of children and adolescents with cancer: a randomized controlled trial. Esc Anna Nery. 2019;24(1): e20190133.

[32] Cheung DSK, Kor PPK, Jones C, Davies N, Moyle W, Chien WT, et al. The use of modified mindfulness-based stress reduction and mindfulness-based cognitive therapy program for family caregivers of people living with dementia: a feasibility study. Asian Nurs Res (Korean Soc Nurs Sci). 2020;14(4): 221–230. doi: 10.1016/j.anr.2020.08.009 32931996

[33] Danucalov MAD, Kozasa EH, Ribas KT, Galduróz JCF, Garcia MC, Verreschi ITN, et al. A yoga and compassion meditation program reduces stress in familial caregivers of Alzheimer’s disease patients. Evid Based Complement Alternat Med. 2013;2013: 513149. doi: 10.1155/2013/513149 23690846 PMC3652205

[34] Varambally S, Vidyendaran S, Sajjanar M, Thirthalli J, Hamza A, Nagendra HR, et al. Yoga-based intervention for caregivers of outpatients with psychosis: a randomized controlled pilot study. Asian J Psychiatr. 2013;6(2): 141–145. doi: 10.1016/j.ajp.2012.09.017 23466111

[35] Butzer B, LoRusso A, Shin SH, Khalsa SBS. Evaluation of yoga for preventing adolescent substance use risk factors in a middle school setting: a preliminary group-randomized controlled trial. J Youth Adolesc. 2017;46(3): 603–632. doi: 10.1007/s10964-016-0513-3 27246653 PMC5133199

[36] Chong CSM, Tsunaka M, Tsang HWH, Chan EP, Cheung WM. Effects of yoga on stress management in healthy adults: a systematic review. Altern Ther Health Med. 2011;17(1): 32–38. 21614942

[37] Wang F, Szabo A. Effects of yoga on stress among healthy adults: a systematic review. Altern Ther Health Med. 2020;26(4): AT6214. 32088671

[38] Adeva-Andany MM, Martínez-Rodríguez J, González-Lucán M, Fernández-Fernández C, Castro-Quintela E. Insulin resistance is a cardiovascular risk factor in humans. Diabetes Metab Syndr. 2019;13(2): 1449–1455. doi: 10.1016/j.dsx.2019.02.023 31336505

[39] Schmiegelow MD, Hedlin H, Stefanick ML, Mackey RH, Allison M, Martin LW, et al. Insulin resistance and risk of cardiovascular disease in postmenopausal women: a cohort study from the Women’s Health Initiative. Circ Cardiovasc Qual Outcomes. 2015;8(3): 309–316. doi: 10.1161/CIRCOUTCOMES.114.001563 25944628

[40] Thacker EL, Psaty BM, McKnight B, Heckbert SR, Longstreth WT Jr, Mukamal KJ, et al. Fasting and post-glucose load measures of insulin resistance and risk of ischemic stroke in older adults. Stroke. 2011;42(12): 3347–3351. doi: 10.1161/STROKEAHA.111.620773 21998054 PMC3226936

[41] Garvey WT, Kwon S, Zheng D, Shaughnessy S, Wallace P, Hutto A, et al. Effects of insulin resistance and type 2 diabetes on lipoprotein subclass particle size and concentration determined by nuclear magnetic resonance. Diabetes. 2003;52(2): 453–462. doi: 10.2337/diabetes.52.2.453 12540621

[42] Goff DC Jr, D’Agostino RB Jr, Haffner SM, Otvos JD. Insulin resistance and adiposity influence lipoprotein size and subclass concentrations. Results from the Insulin Resistance Atherosclerosis Study. Metabolism. 2005;54(2): 264–270. doi: 10.1016/j.metabol.2004.09.002 15690322

[43] Goldberg IJ. Clinical review 124: Diabetic dyslipidemia: causes and consequences. J Clin Endocrinol Metab. 2001;86(3): 965–971. doi: 10.1210/jcem.86.3.7304 11238470

[44] Peckett AJ, Wright DC, Riddell MC. The effects of glucocorticoids on adipose tissue lipid metabolism. Metabolism. 2011;60(11): 1500–1510. doi: 10.1016/j.metabol.2011.06.012 21864867

[45] Harada PHN, Demler OV, Dugani SB, Akinkuolie AO, Moorthy MV, Ridker PM, et al. Lipoprotein insulin resistance score and risk of incident diabetes during extended follow-up of 20 years: The Women’s Health Study. J Clin Lipidol. 2017;11(5): 1257–1267. doi: 10.1016/j.jacl.2017.06.008 28733174 PMC5644504

[46] Flores-Guerrero JL, Connelly MA, Shalaurova I, Gruppen EG, Kieneker LM, Dullaart RPF, et al. Lipoprotein insulin resistance index, a high-throughput measure of insulin resistance, is associated with incident type II diabetes mellitus in the Prevention of Renal and Vascular End-Stage Disease study. J Clin Lipidol. 2019;13(1): 129–137.e1. doi: 10.1016/j.jacl.2018.11.009 30591414

[47] Flores-Guerrero JL, Gruppen EG, Connelly MA, Shalaurova I, Otvos JD, Garcia E, et al. A newly developed diabetes risk index, based on lipoprotein subfractions and branched chain amino acids, is associated with incident type 2 diabetes mellitus in the PREVEND Cohort. J Clin Med. 2020;9(9): 2781. doi: 10.3390/jcm9092781 32867285 PMC7563197

[48] Ellsworth DL, Costantino NS, Blackburn HL, Engler RJM, Kashani M, Vernalis MN. Lifestyle modification interventions differing in intensity and dietary stringency improve insulin resistance through changes in lipoprotein profiles. Obes Sci Pract. 2016;2(3): 282–292. doi: 10.1002/osp4.54 27708845 PMC5043634

[49] Ross LM, Slentz CA, Zidek AM, Huffman KM, Shalaurova I, Otvos JD, et al. Effects of amount, intensity, and mode of exercise training on insulin resistance and type 2 diabetes risk in the STRRIDE randomized trials. Front Physiol. 2021;12: 626142. doi: 10.3389/fphys.2021.626142 33613319 PMC7892901

