# Supplementary material for: Effects of a yoga-based stress reduction intervention on stress, psychological outcomes and cardiometabolic biomarkers in cancer caregivers: A randomized controlled trial
Source: PLoS One. 2022 Nov 10;17(11):e0277009. doi: 10.1371/journal.pone.0277009 (PMC9648784; doi:10.1371/journal.pone.0277009)
Supplement: S1 File — (DOCX) [file pone.0277009.s002.docx]

**Date**: February 14, 2018

**Title:** A randomized controlled trial to determine the effectiveness of a stress reduction intervention in caregivers of allogeneic hematopoietic stem cell transplant (HSCT) recipients.

PRECIS

Millions of Americans provide unpaid care for aging or ill family members and friends. Caregiving for an individual with cancer who is undergoing stem cell transplantation is particularly stressful. The stress of caregiving is associated with many disorders, including sleep disturbances, depression, and anxiety. Although there is good evidence that cancer caregivers experience high levels of stress and stress-related symptoms, few interventions studies have been explored to address this concern in this population.

Stress reduction techniques are important skills for individuals to cope with the stress of cancer caregiving. Mindfulness techniques such as yoga and meditation reduce levels of stress and stress-related symptoms in caregivers. However, many caregivers are reluctant to take time away from the patient to attend to their own health and well-being. Few studies have explored yoga-based stress reduction interventions that can be performed at home or at the patient’s bedside.

Based upon our past research examining stress and stress-related symptoms in stem cell transplant caregivers, this study is designed to determine preliminary effectiveness of a yoga-based stress reduction intervention in reducing levels of stress in caregivers during allogeneic hematopoietic stem cell transplant (HSCT). In addition, this study will explore factors associated with change in caregiver stress and symptoms such as demographics, clinical variables, cardiometabolic and inflammatory markers, and health behaviors.

This study will use a prospective randomized control group design to examine the effects of a six-week yoga-based stress reduction intervention on perceived stress in caregivers of allogeneic HSCT patients. Subjects will be accrued to this protocol if they are a caregiver of an individual undergoing allogeneic HSCT at the Clinical Center, NIH, are > 18 years old, able to read English, stand and sit unassisted, raise arms over head without pain, and able to comprehend the investigational nature of the study. A sample of at least 78 caregivers is needed to adequately evaluate the effectiveness of the intervention.

All caregivers will attend the usual care group education provided at the Clinical Center for transplant caregivers. Caregivers randomized to the intervention group will be scheduled for one session with study personnel to receive a 20 minute audio file of gentle chair yoga poses and guided breath awareness, as well as instructions for performing them, and they will be asked to practice daily. Data, in the form of web-based questionnaires, as well as a physical assessment, demographic interview and blood work will be collected from all study participants at baseline (at or near the time of HSCT) and again at the end of the six-week intervention. There will be no long-term follow-up after the intervention period. Questionnaires include: Caregiver Reaction Assessment, Health-Promoting Lifestyle Profile II, Pittsburgh Sleep Quality Index, Freiburg Mindfulness Scale, PROMIS measures of anxiety, applied cognition, depression, positive affect and well-being, Multidimensional Fatigue Symptom Inventory- Short Form, and NIH Toolbox measures of loneliness, self-efficacy, and perceived stress. Subjects will participate in an exit interview at the end of the study and the interventionist will complete a log, based on subject diaries, that tracks subject practice, and discrepancy between planned and actual session date and time. Quantitative analysis techniques will be used in this study.

Table of Contents

[1.0 STUDY OBJECTIVES & RESEARCH HYPOTHESES 3](#_Toc393800853)

[1.1 Study Objectives & Hypotheses 3](#_Toc393800854)

[1.1.1 Primary Objectives & Hypotheses 3](#_Toc393800855)

[1.2.1 Exploratory Objectives 3](#_Toc393800856)

[2.0 BACKGROUND 3](#_Toc393800857)

[2.1 Stress reduction interventions 6](#_Toc393800858)

[2.2 Scientific Justification 7](#_Toc393800859)

[3.0 STUDY DESIGN 10](#_Toc393800860)

[Figure 2. Participant Flow Progress 11](#_Toc393800861)

[Figure 3: Study Plan 12](#_Toc393800862)

[3.1 Usual Care Education (UCE) 12](#_Toc393800863)

[3.2 Experimental Intervention – Stress reduction intervention 13](#_Toc393800864)

[4.0 ELIGIBILITY ASSESSMENT 14](#_Toc393800865)

[4. 1 Inclusion Criteria 14](#_Toc393800866)

[4.2 Exclusion criteria 14](#_Toc393800867)

[5.0 METHODS 15](#_Toc393800868)

[5.1 Questionnaires: 15](#_Toc393800869)

[5.2 Demographic Interview 18](#_Toc393800870)

[5.3 RESEARCH PARTICIPANT/PATIENT DATA 18](#_Toc393800871)

[5.4 Clinical and Laboratory Assessments 18](#_Toc393800872)

[5.5 Telephone Script and Exit Interview 20](#_Toc393800873)

[5.6 Implementation Log 20](#_Toc393800874)

[6.0 BIOSTATISTICAL CONSIDERATIONS 20](#_Toc393800875)

[6.1 Sample size 20](#_Toc393800876)

[6.2 Statistical Analyses 20](#_Toc393800877)

[7.0 Data Safety and Monitoring Plan 21](#_Toc393800878)

[7.1 Data Safety 21](#_Toc393800879)

[7.2 Monitoring Plan 21](#_Toc393800880)

[8.0 Collecting, Tracking and Disposition of Data & Samples 22](#_Toc393800881)

[8.1 Samples Management 22](#_Toc393800882)

[8.2 Storage 22](#_Toc393800883)

[8.3 Tracking 22](#_Toc393800884)

[8.4 End of Study Procedures 22](#_Toc393800885)

[8.5 Loss or Destruction of Samples 22](#_Toc393800886)

[9.0 REPORTING PROCEDURES 23](#_Toc393800887)

[9.1 Adverse Events 23](#_Toc393800888)

[9.2 Serious Adverse Events 23](#_Toc393800889)

[10.0 HUMAN SUBJECT PROTECTIONS 24](#_Toc393800890)

[10.1 Rationale for Subject Selection 24](#_Toc393800891)

[10.2 Evaluation of Benefits and Risks/Discomforts 25](#_Toc393800892)

[10.3 Consent and Assent Processes and Documents 25](#_Toc393800893)

[10.4 Research Subject Compensation 27](#_Toc393800894)

[11.0 References 38](#_Toc393800895)

**Appendices**

Appendix A: Sequence for Yoga Practice File

Appendix B: Treatment Fidelity

Appendix C: Demographic Interview

Appendix D: Telephone Script and Exit Interview

Appendix E: Subject Practice Log

# STUDY OBJECTIVES & RESEARCH HYPOTHESES

## **Study Objectives & Hypotheses**

## Primary Objectives & Hypotheses

- - - 1. To examine whether HSCT caregivers participating in a stress reduction intervention demonstrate improved levels of perceived stress compared to HSCT caregivers in the control group.
         1. ***Hypothesis:*** The level of perceived stress will differ between HSCT caregivers in the control group and HSCT caregivers who participate in a stress reduction intervention.

## Exploratory Objectives

- - - 1. To examine whether HSCT caregivers participating in a stress reduction intervention demonstrate improvement in certain individual factors (self-efficacy, burden, and mindfulness), psychosocial factors (stress, anxiety, depression, loneliness, positive affect and well-being), behaviors (health behaviors) and symptoms (sleep, fatigue, depression, and cognitive impairment) compared with HSCT caregivers in the control group.
      2. To examine whether HSCT caregivers participating in a stress reduction intervention demonstrate improvement in physiological markers of stress (serum cortisol) and clinical (blood pressure, waist circumference, body weight) and biomarkers of inflammation and cardiometabolic disease (lipids profiles, fasting blood glucose, insulin, IL6, TNF-alpha, hs-CRP) compared with HSCT caregivers in the control group.
      3. To examine whether the amount of yoga practiced by HSCT caregivers in the intervention group contributes to improvement in individual factors, psychosocial factors, behaviors, symptoms, as well as physiological markers of stress and clinical and biomarkers of inflammation and cardiometabolic disease.
      4. To characterize subjects with a significant change in perceived stress or other study outcomes (ie: sleep disturbance, depression, anxiety, loneliness) following the intervention, to generate possible hypotheses for future research.

# 2.0 BACKGROUND

Providing care to a family member or friend is a common yet challenging experience for many in the US, with an estimated 65.7 million people in the US serve as unpaid family caregivers, affecting approximately 36.5 million households [^1^](#_ENREF_1). Having a family member or friend with a diagnosis of cancer is the third leading reason for becoming a caregiver and is widely accepted as stressful [^1^](#_ENREF_1). The stress of caregiving in general can have physical and psychological repercussions that ultimately can lead to increased morbidity and mortality [^2^](#_ENREF_2)^,^[^3^](#_ENREF_3). Spousal caregivers of elder individuals hospitalized for a number of conditions are at increased risk of dying within a year of hospitalization [^4^](#_ENREF_4). In a large-scale (n = 5708) nationally-representative sample of older Americans that controlled for a number of socioeconomic, demographic, and health factors, caregiving predicted the relative risk (RR) of the onset of hypertension in both current (RR = 1.36, 95% confidence interval (CI): 1.01, 1.83) and long-term (RR= 2.26, CI: 1.17, 4.49) caregivers [^5^](#_ENREF_5). Likewise, data from more than 54,000 women in the Nurses’ Health Study showed that caregiving for an ill or disabled spouse for nine or more hours per week placed women at increased risk for cardiovascular disease [^6^](#_ENREF_6). Compared longitudinally with non-caregiving controls, family caregivers demonstrate increased levels of C-reactive protein and tissue-type plasminogen activator antigen [^7^](#_ENREF_7)^,^[^8^](#_ENREF_8), possibly explaining an increased risk for cardiovascular disease in caregivers.

The stress of caring for individuals with cancer is associated with increased inflammation, as caregivers of individuals with glioblastoma have significantly higher levels of IL-6 than control subjects [^9^](#_ENREF_9) Some evidence suggests that the increased risk of mortality seen in caregivers may be a result of the effects of stress, as opposed to the actual act of caregiving [^10^](#_ENREF_10), while others have found physical and psychological benefits to caregiving [^11^](#_ENREF_11)^,^[^12^](#_ENREF_12).

While the debate over the mechanisms for the health risks associated with caregiving continues, an indisputable body of evidence is accumulating across a variety of cancer populations that suggests the burden of caring for a family member with cancer is an extreme stressor. Levels of psychological distress experienced by caregivers are comparable or worse than those reported by their respective cancer patients[^13-16^](#_ENREF_13). Caregivers of cancer patients who were hospitalized and died in the ICU are at heightened risk for PTSD [^17^](#_ENREF_17). Likewise, caregivers of non-cancer ICU patients (e.g. cardiothoracic) had a 30% incidence of PTSD following the death of a loved one in an ICU [^18^](#_ENREF_18)^,^[^19^](#_ENREF_19). Anxiety and depression are frequently reported by cancer caregivers, and these conditions are more likely to worsen as the patient’s health status declines [^20^](#_ENREF_20).

Clearly, the burden of caring for an individual with cancer is stressful. The financial, social, physical and emotional resources required to provide care to a sick individual is known as caregiver burden. According to Applebaum and Breitbart [^21^](#_ENREF_21), a number of factors contribute to caregiver burden, as caregivers who work outside the home, those providing care to more than one individual, and those providing care for longer time periods report higher levels of burden. In addition, caregivers report having to modify their lifestyle to accommodate the care recipient’s needs including: reducing physical activity, decreasing leisure activity including hobbies, and having to modify relationships with friends and family. They report frequently prioritizing their patient’s needs over their own [^22^](#_ENREF_22). The amount and quality of social support reported by caregivers contributes to burden, as poor social integration, lower social support, and negative social interactions are independent predictors of caregiver burden [^23^](#_ENREF_23). The quality of the caregiver/care-recipient relationship (mutuality) also predicts caregiver burden [^24^](#_ENREF_24).

In a comprehensive review of the literature involving 164 research studies examining the burden of caring for individuals with cancer, the researchers identified more than 200 problems and responsibilities associated with cancer caregiving [^25^](#_ENREF_25). The problems identified in the review include: pain; sleep problems, fatigue; digestive difficulties; financial problems; work interference; family conflict; marital strain; anxiety; depression; and feelings of hopelessness, helplessness, loneliness, anger, despair, guilt, and fear.

Sleep problems and high levels of fatigue are two common and burdensome outcomes of cancer caregiving. Sleep problems in caregivers in general have been the focus of much research, and sleep disturbances in caregivers of individuals with dementia and other chronic illnesses are well documented [^26^](#_ENREF_26). Seventeen studies, nearly all descriptive, have examined sleep quality in cancer caregivers, and the majority found at least 40% of cancer caregivers report at least one sleep problem including short sleep duration, nocturnal awakenings, and daytime dysfunction [^26^](#_ENREF_26). HSCT caregivers may be at particular risk, as our own research has shown that nearly 66% of caregivers of individuals undergoing HSCT report poor sleep quality [^27^](#_ENREF_27), yet fewer than 3% of these caregivers report taking sleep medications. Poor sleep quality is not only a problem in and of itself, but poor sleep quality contributes to stress-related symptoms in cancer caregivers including: depression, anger, muscle tension, sympathetic arousal, cognitive disorganization, cardiopulmonary arousal, as well as neurological, gastrointestinal, and upper respiratory symptoms [^28^](#_ENREF_28). Often studied in conjunction with sleep, moderate levels of fatigue were found in a review of the literature of eleven studies examining fatigue in cancer caregivers [^29^](#_ENREF_29). Fatigue in cancer caregivers can mean more than simply feeling tired. Cho et al. [^30^](#_ENREF_30) found that, like sleep disturbances, levels of fatigue are also positively correlated with depression.

While evidence suggests that caring for a sick or disabled individual may lead to increased morbidity and mortality, it is not clear whether this increased risk is a direct result of the burden of caregiving, or whether the experience of caregiving leads one to engage in negative health behaviors, such as decreased healthy activities such as physical activity and stress management, or increased consumption of alcohol, tobacco, or high caloric, nutrient-poor food, that in turn lead to increased morbidity and mortality [^31^](#_ENREF_31). In an Australian study of caregivers of patients with ovarian cancer, more than half reported at least one negative change in health behaviors after their loved one’s diagnosis, including decreased physical activity (42%) and weight gain (35%) [^32^](#_ENREF_32). In a population based comparison of 5,699 informal caregivers and 12,941 non-caregivers, Hoffman, Lee and Mendez-Luck [^33^](#_ENREF_33) found that, while controlling for psychological distress and a number of demographic factors, caregivers had higher odds of negative health behaviors including smoking, and consuming fast food and high sugar drinks.

While it makes intuitive sense that participating in healthy behaviors such as eating healthy food, exercising, and participating in stress reduction activities might help ameliorate the effects of stress associated with cancer caregiving and improve caregivers’ health, few published studies have examined these relationships [^33^](#_ENREF_33). Caregivers report that the demands of caregiving interfere with health promoting behaviors such as exercise and proper nutrition [^32^](#_ENREF_32). In our own research, caregivers of individuals undergoing HSCT report that the health behaviors they practice least often are completing their own preventative health screenings, and participating in physical activity and stress management activities [^31^](#_ENREF_31). These same findings have been found in other studies, [^34^](#_ENREF_34)^,^[^35^](#_ENREF_35) and these researchers concluded that caregivers appear to be willing to participate in activities that provide emotional strength and can be conducted at the patient’s bedside, such as praying or working on improving their relationship with the patient, but they are reluctant to participate in activities that require time away from the patient. Healthy behaviors such as exercise, proper nutrition, and stress reduction appear to matter, as cancer caregivers who participate in fewer healthy behaviors exhibit higher levels of distress and more impaired sleep and fatigue [^36^](#_ENREF_36). Our own research involving caregivers of individuals undergoing HSCT shows that, when examining the importance of individual health behaviors such as nutrition, physical activity, interpersonal relationships, stress management, and spiritual practices, stress management was the only health behaviors that negatively predicted both sleep (p<.01) and fatigue (p<.001) [^27^](#_ENREF_27), underscoring the importance of stress reduction for HSCT caregivers.

## 2.1 Stress reduction interventions

The last decade has seen an increase in intervention studies to reduce stress in caregivers, particularly caregivers of individuals with dementia, and many of these interventions involve yoga or meditation[^37^](#_ENREF_37). Hurley, Patterson, and Cooley [^37^](#_ENREF_37) reviewed eight studies involving meditation-based interventions for dementia caregivers, and found significant reductions (p<.05) in levels of depression in five studies and in burden in three. Mindfulness-Based Stress Reduction (MBSR), a structured yoga and meditation-based intervention typically involving a daylong workshop plus weekly classes, has perhaps been the most common mindfulness intervention, and it has been successful in reducing stress and other symptoms including depression and anxiety in caregivers, but these studies have focused largely on caregivers of the elderly or individuals with dementia, a population of chronic caregivers who have been the focus of primarily home-based interventions [^38-40^](#_ENREF_38). MBSR, as classically taught, requires a substantial time commitment and involves attending classes and workshops away from the patient. However, mindfulness appears to be important, as Kögler et al. [^41^](#_ENREF_41) found mindfulness levels to be highly negatively correlated with distress and poor quality of life in caregivers of individuals in palliative care. Yoga interventions have been effective in reducing stress in dementia caregivers, as 8 weeks of yoga and compassion meditation significantly improved levels of stress, depression, and anxiety, and reduced levels of salivary cortisol in yoga intervention subjects compared to controls [^42^](#_ENREF_42). Mindfulness interventions such as yoga and meditation appear to provide more benefits than just relaxation, as a pilot study involving 12 minutes of daily yogic meditation significantly improved depression and cognitive function in dementia caregivers randomized to receive the yogic meditation intervention (n = 25) compared to those in the control group (n = 20) who listened to relaxation music (p<.05) [^43^](#_ENREF_43); subjects in the intervention group also exhibited significant improvement in telomerase activity compared with controls (p<.05).

Surprisingly few studies have examined the effectiveness of stress reduction interventions in cancer caregivers. In a 2010 meta-analysis of 29 randomized control trials focusing on caregiver outcomes in cancer caregivers, nearly all of the interventions involved psychoeducation, skill training, and/or therapeutic counseling, with the emphasis often placed on improving marital/family relationships or on improving caregivers’ ability to care for the cancer patient [^44^](#_ENREF_44). While a number of these studies may have included certain educational components that addressed stress reduction in the larger context of caregiver self-care, none made stress reduction of the caregiver the primary focus of the intervention, and nearly all focused exclusively on psychosocial outcomes.

Using a convenience sample of 26 patient-caregiver dyads, Lengacher et al. [^45^](#_ENREF_45) examined the effectiveness of a modified, 6-week MBSR intervention on reducing stress and improving physical status in advanced-stage cancer patients and their caregivers. Interestingly, the researchers found significant improvement in levels of stress and anxiety for the patients (p<.05), but not for the caregivers. Because caregivers report so many demands on their time, it is possible that the time commitment of the intervention may have washed out the therapeutic benefit of the intervention. However, in a sample of 60 caregivers of individuals with brain tumors, the large majority (87%) were interested in programs to reduce stress [^46^](#_ENREF_46). While almost half (46%) believed they could commit to a stress reduction program that required biweekly participation, nearly all (90%) preferred stress reduction programs that could be completed in their own homes. Home practice of stress reduction techniques appears to be beneficial, as a recent national study of yoga practitioners showed that it isn’t the number of yoga classes an individual takes that contributes positively to health outcomes [^31^](#_ENREF_31); rather, the amount of time one spends practicing yoga at home predicted health outcomes.

## 2.2 Scientific Justification

In summary, caregiving in general has been shown to be stressful and may even increase one’s risk of morbidity and mortality. Specifically, caregivers appear to be at increased risk for cardiovascular disease [^5^](#_ENREF_5)^,^[^6^](#_ENREF_6), and inflammation is implicated as a potential causal mechanism [^7^](#_ENREF_7). Cancer caregivers report a number of problems associated with caregiving, and our own research has shown that caregivers of individuals undergoing HSCT report significantly higher levels of distress, depression, anxiety, loneliness, sleep disturbances and fatigue than age and gender matched controls [^47^](#_ENREF_47). Engaging in stress reduction activities such as yoga and meditation has been beneficial in reducing stress, burden and symptoms such as depression and anxiety in caregivers in general. Yet nearly all of the stress reduction intervention research has focused on caregivers of the elderly and those with dementia.

Our own research has shown that HSCT caregivers who practice healthy behaviors such as engaging in physical activity and stress management techniques sleep better and have lower levels of fatigue [^27^](#_ENREF_27). Yet caregivers in general and HSCT caregivers in particular, tend not to engage in healthy behaviors that require time away from their patient such as exercise and stress reduction activities. The transplant experience places the caregivers at high risk for significant levels of psychological distress, yet few intervention studies have addressed this concern in this population. Interventions are needed that allow caregivers to experience the benefits of stress reduction without requiring a great deal of time or necessitating the caregiver to leave the patient, whether hospitalized or at home, in order to attend a class.

The primary objective of this study is to examine whether HSCT caregivers participating in a stress reduction intervention demonstrate improved levels of stress compared to HSCT caregivers in the control group. The long term objective of this line of research is the development of behavioral interventions to improve health outcomes in cancer caregivers that can be tested in effectiveness studies, bringing evidence closer to translation into practice. Because caregiving is associated with inflammation and increased risk of cardiovascular disease, a secondary objective will be to explore the effects of the intervention in improving inflammation (IL-6, TNF-alpha, C-reactive protein) and markers of cardiovascular disease risk including components of Metabolic Syndrome, a cluster of symptoms that place an individual at increased risk for coronary artery disease [^48^](#_ENREF_48). Defined by the National Cholesterol Education Program Adult Treatment Panel (NCEP ATP III) as three or more of the following: hyperglycemia (Fasting Blood Glucose ≥ 110 mg/dL), hypertension (BP ≥ 130/85 mmHg), hypertriglyceridemia > 150 mg/dL, low High-Density Lipoprotein Cholesterol (HDL) (< 40 mg/dL in men and < 50 mg/dL in women), and waist circumference > 40 inches in men and > 35 inches in women [^49^](#_ENREF_49). Because plasma lipoproteins such as Apolipoprotein A1 and Apolipoprotein B recently have been shown to predict cardiovascular risk better than the gold standards of HDL and LDL cholesterols [^50^](#_ENREF_50)^,^[^51^](#_ENREF_51), our exploratory objective will include these as well.

This study is guided by a conceptual model proposed by Vitaliano et al. [^52^](#_ENREF_52) (Figure 1). In this model, the individual comes to the caregiver experience with unique characteristics (demographics and self-efficacy). The caregiving experience (measured in this study as caregiver burden), is also unique. This caregiving experience both impacts and is impacted by psychosocial, behavioral, and physiological/clinical factors, as well as symptoms such as sleep disturbance, fatigue, and cognitive impairment.

Figure 1. Conceptual model of factors affecting caregiver health and study measures


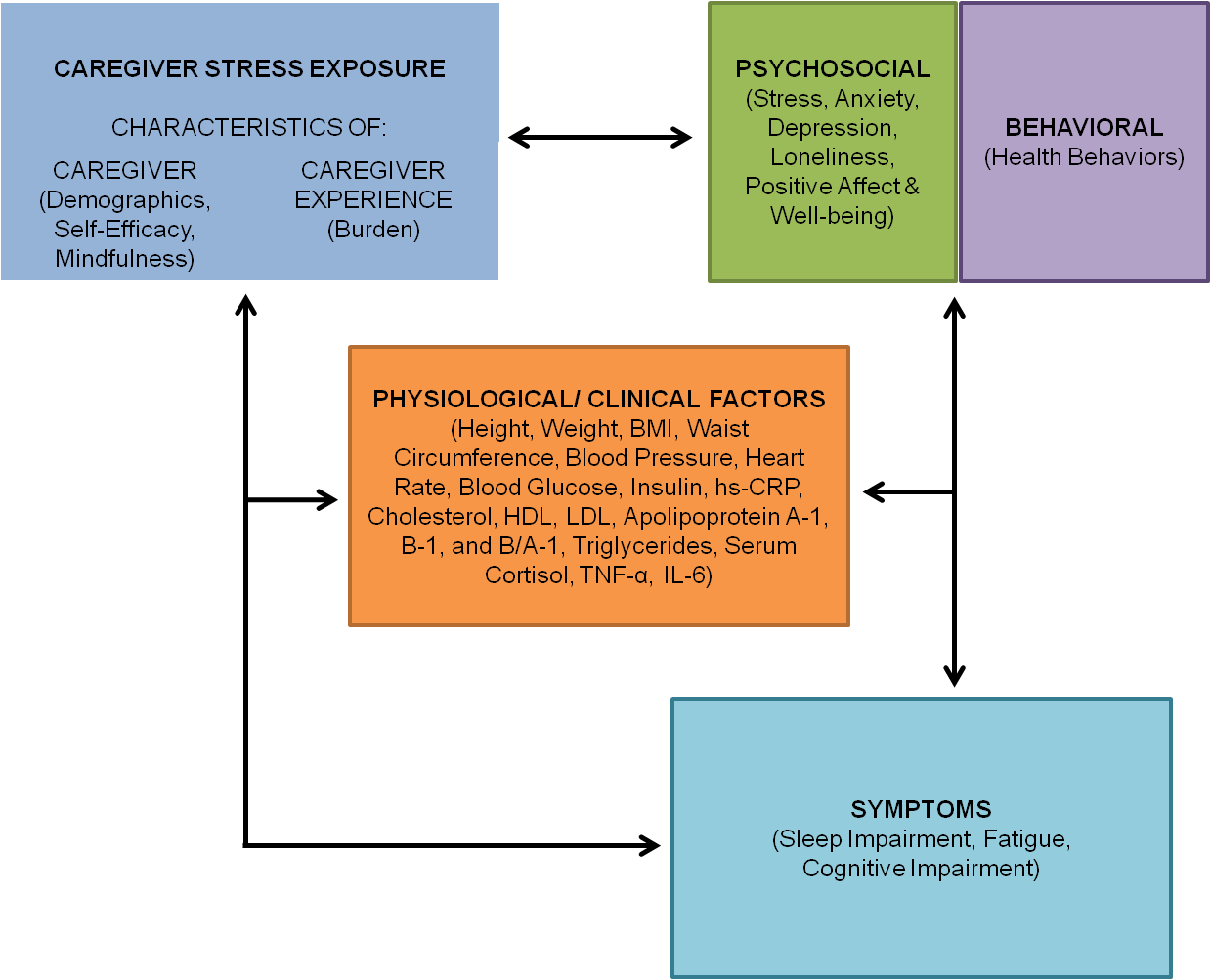


Note: BMI – Body Mass Index, hs-CRP – high sensitivity C-reactive protein, HDL – High Density Lipoprotein, LDL – Low Density Lipoprotein, TNF-α – Tumor Necrosis Factor-α, IL-6 – Interleukin 6.

#

# 3.0 STUDY DESIGN

This study will use a prospective randomized controlled group design to examine the effectiveness of a six-week yoga-based stress reduction intervention on perceived stress of allogeneic HSCT caregivers (Figures 2, 3). Subjects will be accrued to this protocol once they have been identified and are serving as a primary caregiver (defined as ACTIVE caregiver) for an individual planning to undergo an allogeneic HSCT at the Clinical Center, NIH. The patient and caregiver(s) will be approached for participation before the patient starts their transplant conditioning.

All participants will receive usual care education (UCE), which includes transplant specific information for the recipient and the caregiver, as well as caregiver resources. After study enrollment, caregiver subjects will be randomized to either the control group or the intervention group. A permuted block randomization with allocation ratio of 1:1 will be performed by the study statistician. All participants will meet 1:1 with a study investigator to review the UCE materials. Participants in the intervention group will also receive an audio recording of a 20 minute yoga-based stress reduction intervention, as well as instructions on how to complete the intervention; they will be asked to practice the twenty minute audio file daily for the six weeks of the intervention. The six-week length of the intervention, as well as the 20 minute time of the audio file, was selected based upon the length of similar interventions in other caregiver populations, with interventions ranging between four to ten weeks [^37^](#_ENREF_37)^,^[^43^](#_ENREF_43). All study visits will be scheduled around the HSCT recipient’s schedule and visits to minimize burden for the caregiver. The initial visit for all participants will occur prior to the patient’s initial discharge from the hospital and within 7 days of the HSCT infusion (Day 0+7). Subjects in the intervention group will receive a brief phone call at weeks two and four (+/- 7 days) to assess progress and to record practice. All subjects will participate in an exit interview at week six (+/- 7 days). Study questionnaires will be administered to all subjects at baseline and again at week six (+/- 7 days). Completion will take approximately 20 minutes through a web-based system. If the electronic system is unavailable, a hard copy of the questionnaires will be available for completion. Caregivers in either group who complete baseline assessments but are no longer able to return to the clinical center for their week 6 study visit will be given the option to complete the week 6 questionnaires electronically.

## Figure 2. Participant Flow Progress

##
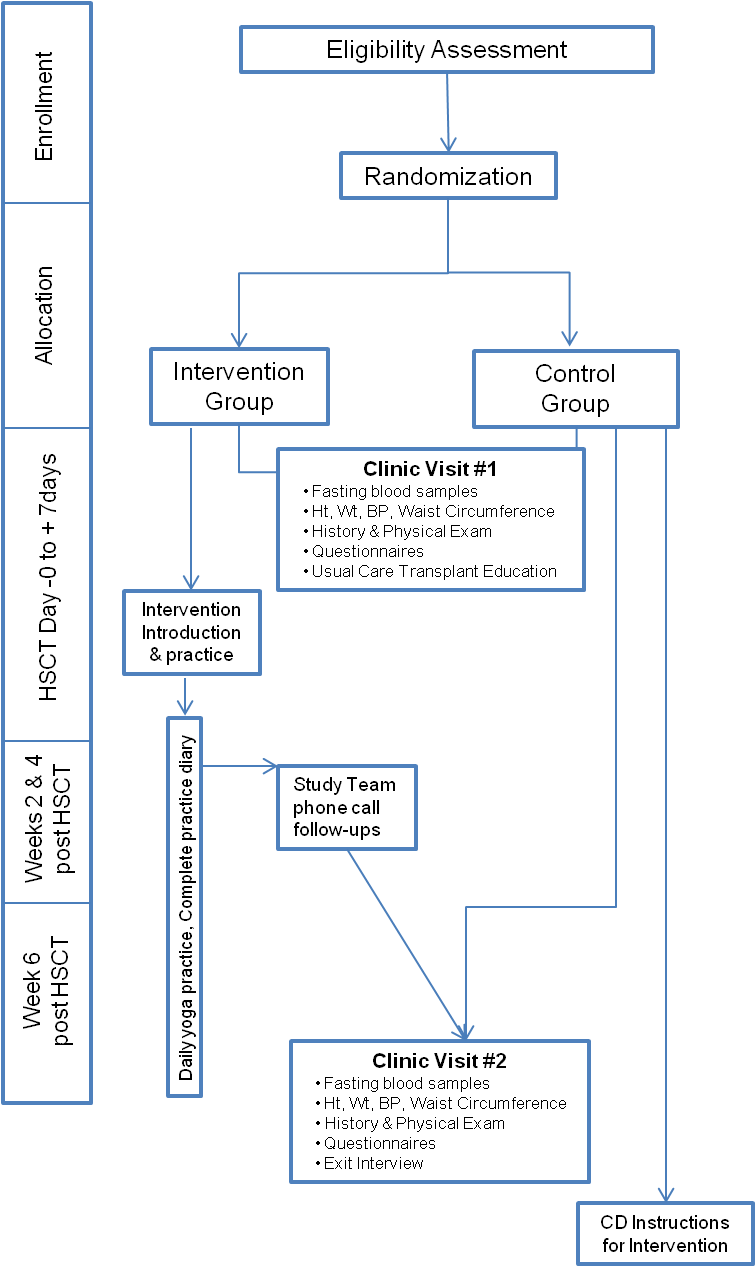


## Figure 3: Study Plan

| Pre-HSCT Cell Infusion (D-0-) | | | Post Transplant Phase | | |
| --- | --- | --- | --- | --- | --- |
| **Time Point** | Baseline Visit & Randomization  (D-0- to +7 days after) | | WK  2  (+/-7 days) | WK  4  (+/-7 days) | WK  6  (+/-7 days) |
|  | Control  Group | Intervention Group | Intervention Group | Intervention Group | All participants |
| Study Activities | | | | | |
| Outpatient Registration | X^a^ | X^a^ |  |  |  |
| Clinical Assessment | X | X |  |  |  |
| Laboratory Samples | X | X |  |  | X |
| Phone call follow-up |  |  | X | X |  |
| Exit Interview |  |  |  |  | X |
| Participant-Reported Psychological & Outcome Measures | | | | | |
| CRA | X | X |  |  | X |
| HPLP-II | X | X |  |  | X |
| PROMIS Measures  Anxiety**  Applied Cognition – General Concerns**  Depression** | X | X |  |  | X |
| NIH Toolbox/Neuro QOL Measures  Loneliness Short Form  Self-Efficacy**  Perceived Stress **  Positive Affect & Well-being | X | X |  |  | X |
| PSQI | X | X |  |  | X |
| MINDFULNESS | X | X |  |  | X |
| MFSI-SF | X | X |  |  | X |
| Demographic Interview | X | X |  |  | X |
| Practice Diary |  | Daily^b^ | | | |
| Clinician Collected Case Report Forms | | | | | |
| Care Recipient Disease & Treatment Characteristics | X | X |  |  | X |

*Note*; CGs: Caregivers; Caregiver Reaction Assessment; HPLP-II: Health Promoting Lifestyle Profile II; PSQI: Pittsburgh Sleep Quality Index; MFSI-SF: Multidimensional Fatigue Symptom Inventory- Short Form; ^a^ Unless previously completed. ^b^ for Intervention Group only; **indicates measure will be administered by computer adaptive test (CAT).

## 3.1 Usual Care Education (UCE)

Participants who are enrolled in allogeneic HSCT research protocols at the Clinical Center receive information from a variety of sources and individuals. Early in the transplant process an institute representative (coordinator) will provide institutional information along with an overview of HSCT from organizations such as the Leukemia and Lymphoma Society, The National Marrow Donor Foundation and Be the Match. Prior to in-patient admission for transplant conditioning, patients and their caregivers receive information specific to the transplant process, complications, monitoring and management, and general tips on caregiving including caregiver specific electronic resources ([www.cc.nih.gov/wecare](http://www.cc.nih.gov/wecare)). Additional information such as medication education is provided to each patient during their admission by the various providers (RN, Pharmacist) involved in their care.

Discharge from the in-patient unit is based on the patient’s clinical recovery (blood counts, nutrition, and infection) as well as the capacity of the patient and the caregiver to manage the complex routine. This includes venous access device (central line care), safe food preparation, transportation, infection prevention strategies, and medication administration. As discharge from the hospital approaches, patients and caregivers are provided with a Transplant Discharge Class to review additional complications and outpatient expectations. In addition, information regarding their new ‘routine’ in the outpatient area is provided. All the documents provided as a component of usual care education are considered expert information. If participants are unable to attend the discharge class, they are given the educational information presented in the class individually by inpatient nursing staff prior to discharge. Receipt of UCE will be documented by the study team.

## 3.2 Experimental Intervention – Stress reduction intervention Appendix A

After baseline measures have been obtained and subjects have been randomized into the intervention and control groups, subjects in the intervention group will meet with the interventionist for approximately 20 minutes to go over instructions for performing the gentle chair yoga and breath work that they will be asked to perform daily using a 20-minute audio recording that they will receive in this meeting. The stress reduction intervention (Appendix A) will be provided to subjects by a trained interventionist. The intervention will be delivered by Alyson Ross, PhD, RN, who is a registered nurse with a master’s degree in psychiatric-mental health nursing, and is a certified Iyengar Yoga Instructor, or by study staff who have received training by Dr. Ross. Participants will then practice the recording one time under the supervision of study staff.

The audio recording was developed by Advanced Iyengar Yoga Instructor, John Schumacher, who has over 35 years of experience teaching yoga and has recorded numerous audiotapes of yoga for home practice. Dr. Ross worked with Mr. Schumacher to develop the intervention, based upon Mr. Schumacher’s years of teaching experience, as well as the research literature that shows the effectiveness of similar yogic breathing exercises in reducing levels of perceived stress [^53^](#_ENREF_53) and similar chair-based yoga interventions in improving levels of depression, anxiety, quality of life, and well-being in elderly populations [^54^](#_ENREF_54)^,^[^55^](#_ENREF_55). The 20-minute audio recording will lead participants through a series of gentle stretching and breathing exercises [^53-55^](#_ENREF_53). The first few poses will be performed standing, using the wall for support. The remainder of the audio file will consist of gentle seated yoga poses (stretches and twists) that are performed in a chair, as well as guided breathing exercises that focus primarily on slowing and quieting the inhalations and exhalations.

Small (<5 GB) audio players will be purchased and given to subjects in the intervention group. These devices will be preloaded with the 20-minute audio file of the stress reduction intervention. Upon completion of the study, control group subjects will also receive an audio player with the intervention recording and no subjects will be asked to return their device.

Subjects in the intervention group will be asked to practice the 20-minute audio recording daily. They will be asked to maintain a diary of practice, and study staff will contact them by phone at weeks two and four for a brief call to answer questions and to record practice days (this same information will be obtained in person at the exit interview). Subjects in the control group will be offered a complementary audio file of the same stress reduction intervention received by the intervention group, along with written instructions regarding how to perform the exercises, at the conclusion of their participation in the study.

The PI will take several measures to ensure the integrity and consistency of the intervention across subjects, and these measures are described in the Treatment Fidelity document in Appendix B. The interventionist is a certified Iyengar yoga instructor, and received training for the stress reduction component of the intervention from Advanced Iyengar Yoga instructor John Schumacher, who created the practice audio files for study participants. Interventionists will review instructions for completing the gentle chair yoga poses and breathing exercises with subjects in the intervention group, and will have all subjects demonstrate understanding of how to perform these in a 1:1 initial session. Lastly, random audits of the intervention delivery by the study team through observation will assure that the intervention is being delivered as designed. In addition to practice diaries to record the amount of practice performed by the intervention group, study staff will contact members of the intervention group every two weeks during the intervention to verify amount of practice. Study staff will maintain an electronic log of all in-person and telephone contact with subjects and the documentation will be reviewed by the study team monthly when subjects are actively enrolled.

# 4.0 ELIGIBILITY ASSESSMENT

## 4. 1 Inclusion Criteria

- Age > 18 years old
- Ability to comprehend the investigational nature of the study
- Able to read and speak English
- Agrees to participate in the study
- Able to lift arms over head without pain
- Able to sit and stand from a seated position unassisted
- Intends to serve as an active caregiver* for a patient undergoing their 1^st^ allogeneic HSCT at the NIH Clinical Center

## Exclusion criteria

- - - Age < 18 years old
    - Pregnant or lactating women
    - Inability to comprehend investigational nature of study
    - Inability to provide informed consent
    - Unable to read and speak English
    - Does not agree to participate in study or follow study design
    - Serving as a paid caregiver for any individual
    - Regular practitioner of yoga, meditation or other mind-body practice (definition: taking classes or practicing at least weekly for at least 2 of the past 6 months).
    - Physical function limitations that would interfere with intervention

Caregiver participants will be excluded from the biomarker analysis if they have had:

- - - Glucocorticosteroid treatment in the last 2 months
    - Have a recent (within the last 2 months) acute illness or injury
    - Have served as a stem cell transplant donor or have taken filgastrim

*****If more than one caregiver is planned for the transplant recipient during the transplant phase, only 1 caregiver will be eligible to participate in the study. An active caregiver is defined as someone who lives with or provides care regularly for the HSCT recipient during the 6 week study period. Caregivers will be categorized as to whether they are the sole caregiver versus one of multiple caregivers.

# 5.0 METHODS

## 5.1 Questionnaires:

5.1.1 Caregiver Reaction Assessment

5.1.2 NIH Toolbox/Neuro QOL Measures

5.1.3 Health-Promoting Lifestyle Profile II

5.1.4 PROMIS^®^ Measures

5.1.5 Pittsburgh Sleep Quality Index

5.1.6 Freiburg Mindfulness Inventory-Short form

5.1.7 Multidimensional Fatigue Symptom Inventory- Short Form (MFSI-SF)

**5.1.1 Caregiver Reaction Assessment (CRA)**

The CRA is designed to assess the positive and negative effects of caregiving for persons providing care to patients with chronic illnesses. The measure consists of 24 items that comprise five subscales including: caregiver esteem, lack of family support, impact on finances, impact on schedule, and impact on health. Participants indicate their agreement with the given statements on a 5-point Likert Scale ranging from “strongly disagree” (1) to “strongly agree” (5) with higher scores denoting a greater impact of the attribute for the caregiver.

In addition to a sample of caregivers of Alzheimer’s patients, the initial testing of the scale included an evaluation of its effectiveness among a sample of 276 caregivers of cancer patients undergoing active treatment for a new or recurring solid tumor or lymphoma [^56^](#_ENREF_56). Internal consistency for the subscales ranged in correlation from 0.80 to 0.91, indicating high reliability. Construct validity has been supported through strong correlations with the Center for Epidemiologic Studies Depression Scale (CES-D Depression Scale) [^57^](#_ENREF_57) and the Activities of Daily Living Dependency Scale (ADL Dependency Scale) [^58^](#_ENREF_58).

**5.1.2 NIH Toolbox and Neuro QOL Measures**

NIH Toolbox and Neuro QOL are a multidimensional set of brief measures assessing cognitive and emotional function from adults. Stress and Self-Efficacy focus on individual perceptions about the nature of events and their relationship to the perceived coping resources of an individual. In general, psychological stress is said to occur when an individual perceives that environmental or internal demands that are personally meaningful exceed his or her adaptive capacity. Operationally, psychological stress is defined by individual reports of adaptive capacity overload. However, it has been defined by the presence of a stressor deemed taxing by consensus, for example, the death of a close other. If the electronic system is unavailable, and the three questionnaires normally given as computer-adaptive tests**are given as hard copy questionnaires, they will be scored in accordance to the comparable short form (fixed item questionnaire). Reliability for this scale has been consistently high with Cronbach’s alphas ranging from 0.91 to 0.95 [^59^](#_ENREF_59).

- Loneliness Short Form
- Self-Efficacy**
- Perceived Stress **
- Positive Affect & Well-being**

**5.1.3 Health-Promoting Lifestyle Profile II (HPLP-II)**

Walker and Hill-Plorecky [^60^](#_ENREF_60)^,^[^61^](#_ENREF_61) developed the HPLP-II, which is a self-administered 52-item instrument that measures the frequency of self-reported healthy behaviors. It consists of 6 subscales: physical activity, spiritual growth, health responsibility, interpersonal relations, nutrition, and stress management. This is a 4-point Likert type scale with responses ranging from 1 (never) to 4 (routinely), and the possible scores ranging from 52 to 208. The higher scores indicate the more frequent engagement in health behaviors. Callaghan [^62^](#_ENREF_62) reported the following Cronbach’s alpha coefficients of internal consistency reliability: total scale 0.93, health responsibility 0.83, physical activity 0.87, nutrition 0.76, spiritual growth 0.84, interpersonal relations 0.82, and stress management 0.75.

**5.1.4 PROMIS^®^ Measures**

Patient-Reported Outcomes Measurement Information System (PROMIS^®^), is an initiative based on an NIH grant to establish and provide the public a free, valid and reliable (Cronbach’s alpha ranging from 0.76-.96) measures for a variety constructs [^63^](#_ENREF_63)^,^[^64^](#_ENREF_64). The PROMIS^®^ item databanks have been tested for reliability and comparability to more established measures of these same content areas by NIH scientists and select US academic institutions using Item-Response Theory [^65^](#_ENREF_65). If the electronic system is unavailable, and the three questionnaires normally given as computer-adaptive tests** are given as hard copy questionnaires, they will be scored in accordance to the comparable short form (fixed item questionnaire).

- Anxiety**
- Applied Cognition – General Concerns**
- Depression**

**5.1.5 Pittsburgh Sleep Quality Index (PSQI)**

The PSQI is an 18 item self report measure of subjective sleep quality [^66^](#_ENREF_66). Clinical information about sleep parasomnias (such as snoring, restless legs, and sleep apnea) is assessed by the bed partner in 5 additional questions that are not used in the scoring. Items for the PSQI were generated from a review of existing questionnaires and from clinical experience, and the instrument underwent 18 months of initial field testing [^66^](#_ENREF_66). Response format is on a four point scale, with 0 = no difficulty and 3 = severe difficulty. The response frame is over the past month; although a modification of the instrument assessing subjective sleep quality over the past week has also been utilized[^67^](#_ENREF_67). The PSQI takes approximately 5-10 minutes to complete. Responses to the 18 items are grouped into seven equally weighted component scores. The component scores measure the domains of subjective sleep quality, sleep latency, sleep duration, habitual sleep efficiency, sleep disturbances, use of sleeping medications, and daytime dysfunction. The 7 component scores can be summed to produce a global score ranging from 0-21. Higher scores indicate more severe complaints and worse sleep quality [^66^](#_ENREF_66). There is strong support for the psychometric properties of the PSQI in a variety of clinical populations and in research [^67-78^](#_ENREF_67). Internal consistency reliability in samples of patients with cancer ranged from 0.70 to 0.81 [^67^](#_ENREF_67)^,^[^70^](#_ENREF_70). Relative to construct validity, a global PSQI score >5 provided a sensitive and specific measure of poor sleep quality compared with clinical and polysomnographic measures [^66^](#_ENREF_66).

**5.1.6 Freiburg Mindfulness Inventory-Short Form**

The Freiburg Mindfulness Inventory-short form is a 14-item self- administered questionnaire that is designed to measure the concept of mindfulness, defined as an awareness and friendly acceptance of one’s mental processes including sensations, perceptions, cognitions, and affects [^79^](#_ENREF_79). Designed to be used in the general population with individuals not necessarily familiar with mindfulness or Buddhist concepts, the short form was derived from the 30-item Freiburg Mindfulness Scale, and it has good reliability and validity compared to the original scale (Cronbach’s alpha = .86) [^79^](#_ENREF_79). Higher scores indicate higher levels of mindfulness.

**5.1.7 Multidimensional Fatigue Symptom Inventory- Short Form (MFSI-SF)**

The MFSI-SF is a 5 point Likert-scale used to assess various dimensions of fatigue. Though originally created for use with cancer patients, the measure have been effectively used with other populations due to its non-disease specific characteristic[^80^](#_ENREF_80). Among the populations that have utilized this scale include patients with various cancers, orofacial pain populations, and healthy subjects[^81-83^](#_ENREF_81).

The MFSI-SF contains 30 items derived from the original 82-item MFSI. Subjects are asked to rate their level of fatigue in the past week on a scale ranging from 0 (not at all) to 4 (extremely). A total of five fatigue subscales make up the measure including: general, physical, vigor, emotional, and mental. Reliability and validity have been established in multiple studies. The original study indicated significant internal reliability for each of the subscales with alpha coefficients ranging from 0.87 to 0.96[^80^](#_ENREF_80). In accordance, validity is significantly high. To demonstrate concurrent validity, the MSFI-SF was measured against the Fatigue Symptom Inventory (FSI) and the SF-36 Vitality Scale and convergent validity was established using the SF-36 Physical Composite score.

## 5.2 Demographic Interview Appendix C

A demographic interview will assess basic demographic information about the caregiver subject including employment status, living arrangements, caregiving hours, concurrent caregiving obligations, whether the caregiver is a sole caregiver or one of multiple caregivers, quantitative alcohol and tobacco consumption, and occupation.

## 5.3 RESEARCH PARTICIPANT/PATIENT DATA

**5.3.1. Transplant recipient data:**

A waiver of consent (see section 10.3.1) is requested to obtain non-research clinical data on the HSCT patient (care recipient) who is receiving HSCT treatment at the NIH and is the associated patient for whom the enrolled caregiver is providing care. This information will be collected from the Clinical Research Information System (CRIS record only) on the patient and includes: disease, stage of disease, date of treatment, type of treatment, treatment protocol, inpatient hospital length of stay, total hospital days during study period, number of readmissions, frequency of outpatient visits for clinical care and total days as outpatient during the caregiver subject’s participation in this study. These data will be used to characterize the caregiver’s experience.

**5.3.2 Caregiver participant data**

A complete medical history, physical exam, review of current medications, assessment of recent stressful life events, and ECOG performance status. Medications will be classified into several categories to include: diabetic, cardiovascular, central nervous system active medications, non-steroidal anti-inflammatory, and other. Additionally data collected will be used to determine their co-morbidity index score and will be calculated using the Charleson Co-morbidity index score [^84^](#_ENREF_84). The Charleson Co-morbidity index score determines an individual’s 10 year risk for mortality by assigning a numerical score to one’s co-morbid conditions to determine a combine score for overall risk for mortality.

## 5.4 Clinical and Laboratory Assessments

**5.4.1 Clinical Assessments**

A complete medical history, physical exam and review of current medications, smoking and alcohol consumption, measurement of height, weight, blood pressure, heart rate, and waist circumference. Participants will be weighed in light clothing without shoes to the nearest 0.1 kg and their heights, in centimeters (cm). Body mass index (BMI) will be calculated using the following calculation:

$$\text{BMI=weight}\frac{\text{kg}}{\left[ \text{height }\left( \text{cm} \right) \right]\text{2}}$$

Waist circumference will be measured at the level of the umbilicus with the participant in mid-expiratory position. Blood pressure, measured by Dinamap^®^ Pro 100-400v2 electronic blood pressure machine or by manual sphygmomanometer on either the right or left upper arm of the participant following a 5-minute rest period in a sitting position. Clinic staff obtaining measurements on study participants will be educated on the techniques to obtain measurements for this study.

The presence of metabolic syndrome will be determined based on the NCEP ATP III [^48^](#_ENREF_48). Subjects will be categorized as ‘positive’ if three or more of the following are present: hyperglycemia (fasting blood glucose ≥ 110 mg/dL), hypertension (BP≥130/85 mmHg), hypertriglyceridemia (triglycerides>150 mg/dL), low High-Density Lipoprotein Cholesterol (HDL<40 mg/dL in men and <50 mg/dL in women), and waist circumference >40 inches in men and >35 inches in women. If three or more are not present, subjects will be categorized as ‘negative’ relative to metabolic syndrome.

**5.4.2 Laboratory Samples**

Approximately 4 tablespoons of venous blood will be collected by either a certified phlebotomist or registered nurse for the following cardiovascular, metabolic and inflammatory markers including but not limited to:

*Cardiovascular and Metabolic biomarkers**

Lipid panel (such as cholesterol, HDL, LDL)

NMR lipid analysis

Apolipoprotein panel (Apo A-1, Apo B, Apo B/Apo A-1)

Fasting blood glucose

Fasting insulin

*Stress markers*

Cortisol: Serum

*Inflammatory Markers*

TNF-α**

IL-6**

hs-CRP

**Note:** *=Participants will need to be fasting for 10 – 12 hours pre-specimen collection; **=Research lab samples will be processed by Dr. Richard Child’s lab.

***Instructions for blood sampling:***

Study participants will be instructed verbally and given the following written instructions to prepare for blood sampling. Please report to the outpatient phlebotomy area for blood work in the morning. Please do not eat for 10 – 12 hours before blood work collection.

**5.4.3 Laboratory Sample Routing**

The following samples will be sent for processing in the **Clinical Center Department of Laboratory Medicine:**

Lipid panel, NMR analysis**,** Apolipoprotein panel (Apo A-1, Apo B, Apo B/Apo A-1), serum cortisol, hs-CRP.

The following laboratory samples will be sent for processing to **Dr. Richard Child’s research lab, building 10-CRC, room 5-5272:**

TNF-α, IL-6.

##

## 5.5 Telephone Script and Exit Interview-Appendix J

The Intervention Group will participate in a brief telephone follow-up at weeks two and four, as well an exit interview at week six. The control group will participate in an exit interview at week six. The length of the interview is projected to be from 10 - 15 minutes.

## 5.6 Implementation Log- Appendix E

A study implementation log will be used to track subject participation in UCE and the details of the initial 1:1 intervention session.

# 6.0 BIOSTATISTICAL CONSIDERATIONS

## 6.1 Sample size

The study is a randomized controlled trial with two groups: stress reduction and control (standard education) using permuted block randomization with allocation ratio of 1:1. The primary objective of this study is to investigate whether HSCT caregivers in the stress reduction group demonstrate improved levels of stress compared to those in the control group. Power analysis was performed based on the primary objective only. We used a two-sided hypothesis that the PSS level will differ between the intervention group and the control group. We assume an exchangeable correlation structure of PSS. Based on an effect size of 0.6 [^40^](#_ENREF_40)^,^[^85^](#_ENREF_85) and correlation of 0.80 between two time points from our biomarker study, with 80% power and type I error of 0.05, in order to detect a difference between the stress reduction group and the control group, we will need 39 participants in each group and a total sample size of 78 [^86^](#_ENREF_86).

## 6.2 Statistical Analyses

Initial data analysis will consist of examining the frequency distributions for all variables at both time points and computing descriptive statistics appropriate for the level of measurement (e.g., mean and standard deviation for interval level data, median for ordinal level data). Linear mixed repeated measures analysis will be used to analyze the effect of intervention on the primary outcome of perceived stress. The primary analysis will be based on intention-to-treat, which will include all of the randomized subjects. The last observation carried forward method will be used to impute missing data. However, if an enrolled subject comes off study before being randomized, they will be replaced. A sensitivity analysis will be performed for all subjects with complete data at both time points.

The effect of intervention on the exploratory outcome variables as described in the objective section will also be examined by linear mixed models. Any demographic covariates will be included in the model if significantly associated with the outcome and significantly different between the groups. To examine the effect of practice variation on the study outcomes, practice time will be included in the linear mixed models for the intervention group only. Aikake information criterion and the Bayesian information criterion will be used to select best fitting models. Appropriate data transformation technique will be used if necessary.

If no significant differences are found between groups, the responders with clinically meaningful changes in the intervention group will then be characterized. No minimal clinically important differences have been established for our primary outcome, the PSS, therefore, we will use the method proposed by Norman, Sloan, and Wyrwich (2003) of accepting changes in .5 standard deviation as clinically important [^87^](#_ENREF_87). Responders and non-responders will be compared on the basis of their demographic characteristics such as gender and age.

In addition to the primary analysis outlined above, exploratory hypotheses generating analyses will be performed to examine the relationships among caregiver factors. Methods of correlation and regression analysis will be used to evaluate the relationships between outcomes of interest and study variables. All inferential tests will be conducted at the .05 level of significance.

# 7.0 Data Safety and Monitoring Plan

## 7.1 Data Safety

Given the research mandate of the NIH, patient data including the results of testing and responses to treatment will be entered into an NIH-authorized and controlled research database. Any future research use will occur only after appropriate review of human subject protections and approval by the IRB or the NIH Office of Human Subjects Research (OHSR).

All human subjects’ personally identifiable information (PII) as defined in accordance to the Health Insurance Portability and Accountability will be separated from individual subject data. Protocol eligibility and consent verification will be tracked and separated from individual subject data. Primary data obtained during the conduct of the protocol will be kept in secure network drives that comply with NIH security standards. Primary and final analyzed data will have identifiers so that research data can be attributed to an individual human subject participant required for subject identification, e.g., study-specific identifying number (SSPIN) generated by Principal Investigator and/or Research Nurse for subject identification. The protocol and all primary and analyzed data will be stored in the NIH Clinical Center’s secure network I drive***.***

## 7.2 Monitoring Plan

***Principal Investigator:*** Accrual and safety data will be monitored by the principal investigator who will provide oversight to the conduct of this study. The protocol will be continuously evaluated for any unusual or unpredicted complications that occur.

***NHLBI IRB:*** Prior to implementation of this study, the protocol, proposed consent and safety data will be reviewed and approved by the properly constituted Institutional Review Board (IRB), operating according to 45 CFR 46. This committee will approve all amendments to the protocol or informed consent and conduct continuing annual review so long as the protocol is open to accrual or sample and/or data analysis continues.

# 8.0 Collecting, Tracking and Disposition of Data & Samples

## 8.1 Samples Management

During the course of participating in this study survey, clinical data, and biological specimens (i.e. blood) will be collected as described in Figures 2 and 3. These data will be used as outlined in the study objectives. Data from all subjects removed from the study will be included in the intention-to-treat analysis.

## 8.2 Storage

Clinical data will be collected using subjects’ names in the source document. However, clinical report forms, paper questionnaires, and research samples will be coded. Research specimens will be stored in Dr. Childs’ lab according to standard procedures. The key to the code, as well as all results of research tests and data (including subject survey responses) will be maintained in a secure network password-protected database housed on a dedicated in-house server protected according to federal standards. The Principal Investigator (Alyson Ross, RN, PhD) will be responsible for overseeing entry of the data into a password protected electronic system and ensuring data accuracy, consistency and timeliness. The principal investigator, associate investigators/research nurses and/or a contracted data manager will assist with the data management efforts. Data will be stored and in locked cabinets and a password protected database until it is no longer of scientific value. Only the Principal Investigator and designated Associate Investigators have access to identified data in the database. Any printed records with identifier information will be kept in a locked file cabinet within a secure file cabinet of the PI.

## 8.3 Tracking

Investigators will be responsible for administering the questionnaires through the web-based system. If a hard-copy of the survey is administered, the investigators will collect the questionnaires from the subject and ensure the delivery of the data to the secure office of the principal investigator. Blood samples will be ordered and tracked through the CRIS Research Screens.  Should a CRIS screen not be available, the NIH form 2803-1 will be completed and will accompany the specimen and be filed in the medical record. Data will not be sent outside NIH without IRB notification and an executed MTA.

## 8.4 End of Study Procedures

Samples from consenting subjects will be stored until they are no longer of scientific value or if a subject withdraws consent for their continued use, at which time they will be destroyed.

## 8.5 Loss or Destruction of Samples

If we become aware that a major breach in our plan for tracking and storage of samples/data has occurred, the IRB will be notified.

# 9.0 REPORTING PROCEDURES

Adverse events, Protocol deviations (PDs), Unanticipated problems (UP), and serious adverse events (SAEs), are defined in NIH HRPP SOP 16 (“Reporting Requirements for Unanticipated Problems, Adverse Events and Protocol Deviations.”). All adverse events occurring during the study, including those observed by or reported to the research team, will be recorded. Serious UPs, and serious PDs, will be reported to the IRB and Clinical Director as soon as possible but not more than 7 days after the PI first learns of the event. Not serious UPs will be reported to the IRB and Clinical Director as soon as possible but not more than 14 days after the PI first learns of the event. Not serious PDs will be reported to the IRB as soon as possible but not more than 14 days after the PI first learns of the event. In addition, SAEs that do not meet the criteria of UP will be reported to the IRB Chair and Clinical Director within 14 days of learning of the event using the SAE form in PTMS.

Expected or non-serious adverse events will be reported at the time of continuing review and are defined in section 9.1. The principal investigator will provide continuous, close monitoring of data and side effects, and adverse events to identify trends. The principal investigator will be responsible for revising the protocol as needed to maintain safety.

## 9.1 Adverse Events

Adverse events reported under this protocol will be limited to those events which are possibly, probably or definitely related to the research described in this protocol. An serious adverse event is defined as any untoward medical occurrences that 1) result in death, 2) are life-threatening, 3) require hospitalization, 4) cause persistent or significant disability /incapacity, 5) result in congenital anomalies or birth defects, 6) are other conditions which in the judgment of the investigators represent significant hazards. All serious adverse events will be reported to the IRB within 7 days for death or life threatening adverse event and within 15 days for all others.

The principal investigator will report events to the IRB promptly in accordance with NIH Human Research Protection Program Standard Operating Procedure #16 (Reporting Requirements for Unanticipated Problems, Adverse Events and Protocol Deviations). The principal investigator will provide continuous, close monitoring of data and side effects and adverse events to identify trends. The principal investigator will be responsible for revising the protocol as needed to maintain safety. The IRB will review adverse events annually at time of continuing review to also evaluate trends and will require follow up plans from the principal investigator whenever a trend is identified.

The anticipated adverse consequences of participation in this protocol are the inconvenience of time required for study procedures (i.e. stress reduction intervention practice), venipuncture, possible emotional distress related to questions that relate to the affective state of the subject, and minor muscle soreness or strain from the intervention. Because of the gentle nature of the intervention, it is unlikely to cause any adverse events.

## 9.2 Serious Adverse Events

Serious adverse events are defined by federal regulations and include events which:

- Are fatal or life threatening
- Result in significant or persistent disability
- Require or prolong inpatient hospitalization
- Result in a congenital anomaly or neoplasm
- Result from an overdose
- Are other conditions which in the judgment of the PI represents a significant hazard

It is not anticipated that any SAEs are expected for subjects participating in this study. However, any reports of serious psychological distress or report of muscle soreness or strain that results in severe impairments in function in subjects will be addressed and reported immediately. These concerns will be reported to the principal investigator and the MAI. If the clinician agrees with the assessment, a referral to a community provider will be completed. Appropriate referrals for immediate assistance in their home community will be made in collaboration with the Department of Social Work.

Serious adverse events **that are determined to be possibly, probably or definitely associated with any study activities** will be reported to the Principal Investigator of this study:Alyson Ross, RN, PhD, Bldg10, Room 2B07, Phone 301-451-8338.

Serious adverse events will be reported to the IRB using the NHLBI IRB Serious Adverse Event Form. The NHLBI IRB will receive a written report of the SAE within 7 days of a death or 15 days of any serious adverse events as outlined in the Interim Guidelines for Adverse Event Reporting.

If the serious adverse event is thought to be due to the experimental component of the protocol, accession to the protocol will be stopped until a full discussion with the IRB has been held. Serious adverse events that are deemed possibly, probably or definitely related to the caregivers baseline health status, undiagnosed conditions, or concurrent therapies will not be reported.

# 10.0 HUMAN SUBJECT PROTECTIONS

## 10.1 Rationale for Subject Selection

The following populations will be excluded from participation in this study and are in accordance with the following NIH HRPP SOPs: 14A Research Involving Vulnerable Subjects, 14B Research Involving Pregnant Women, Human Fetuses and Neonates, 14D Research Involving Children, and 14E Research Involving Adults who are or may be Unable to Consent. Subjects who do not speak and read English will be excluded because the intervention requires the use of an audio-file that is not available in other languages at this time. Pregnant and lactating women are excluded from participation due to the inherent physiological changes that are associated with these circumstances. Children and those unable to provide informed consent or do not understand the investigational nature of the study are excluded because the intervention requires a level of self-responsibility that is likely a problem with these populations.

In our previous study of transplant caregivers, there was a 10% attrition rate for caregivers related to patient death and other complicating factors. Patients receiving an allogeneic transplantation are quite ill and may die during the study period at which point the caregiver(s) would be removed from study. Because the death of a loved one may result in additional stress and the intervention may be helpful in reducing stress, subjects in the intervention group who are removed from the study will be encouraged to keep the audio file and continue practicing the exercises if they find them beneficial. Individuals in the control group who are removed from the study will be given the option of receiving the CD and instructions for the audio file, should they desire to do so. Because the burden of the study may be a deterrent for caregiver participation, we are including compensation for their participation and inconvenience for this study.

## 10.2 Evaluation of Benefits and Risks/Discomforts

There is no greater than minimal risk associated with this study. The time to participate in the initial session for subjects in the intervention group is approximately 30-45 minutes. The time to complete the physical assessment, blood draws, and questionnaires is approximately 60 minutes.

The risks from gentle chair yoga are minimal. While unlikely, minor muscle soreness and strains could potentially occur, particularly in individuals who are sedentary or not used to stretching. The potential emotional distress related to questionnaire completion is not likely to be greater than that experienced by the participants while dealing with their patient’s clinical transplant care and recovery. To monitor potential respondent burden, an investigator will be available to talk with the participant should questions or issues arise during online questionnaire completion.

**10.2.1** Standard Clinical Results

The results of routine clinical assessment and laboratory finding (CLIA approved) will be returned to subjects by a qualified health care provider on the research team at the end of the study. However, any results that are found to be abnormal will be reviewed by the PI and an appropriate medical provider with follow-up plan presented to the subject with their findings. If there is a concern that an abnormal finding is incorrect, the clinical tests will be repeated. If the value remains abnormal, the participant will be informed of the abnormality and referred to their personal physician for follow-up in addition to the medical consultation outlined above.

## 10.3 Consent and Assent Processes and Documents

The principal investigator or designated associate investigators (section 10.3.1) will obtain informed consent. If the subject is not able to articulate understanding of the study, they will not be enrolled. An assent process is not required since children under the age of 18 will not be included. If the participant is known or reports that they are an NIH employee and is interested in participating in this study, they will be referred to the NIH Manual Chapter 2300-630-3, which details the NIH Leave Policy for NIH employees who wish to participate in an NIH biomedical research studies. Additionally, NIH HRPP SOP 14F, Research Involving NIH Staff as Subjects, will be reviewed and Appendix B: NIH Information Sheet on Staff Research Participation will be given to the subject prior to signing the consent document and participating in the study.

**10.3.1** Consent Process, Associate Investigators

The consent will be obtained by the principal investigator or any of the following associate investigators: Sharon L. Flynn, MS, RN, CRNP-BC, OCN^®^, Brenda Roberson, RN, BSN, OCN^®,^ Alyson Ross, RN, PhD; Nonniekaye Shelburne, CRNP, MS, AOCN^®^; Leslie Wehrlen, RN, BSN, OCN^®^ and are indicated by a “*” on the protocol investigator list.

Consenting Investigators will provide each potential participant:

- A brief explanation of the study emphasizing the amount of time involved completing study questionnaires and study visits are incorporated.
- Review biological samples intended for collection and schedule.
- Provision of written consent form time for independent review.
- Verbal consenting process beginning with ‘test’ questions to assess comprehension of important study points outlined in the written consent form.

Potential subjects will be encouraged to ask questions prior to enrolling in this study. The subjects will be reassured that participation in this study is entirely voluntary. Subjects will be informed that their decision to participate or decline in this study will have no impact on their participation in other clinical protocols at the NIH or the transplant recipients’ care. Additionally, once enrolled, the subject has the right to withdraw from this study at any time. Furthermore, if the subject’s HSCT recipient expires during the study period, the caregiver subject will be removed from study on the HSCT recipient’s date of death.

As mentioned in section 5.3.1, non-research clinical information will be collected on the HSCT patient (care receipt) to characterize the caregiver’s experience. A request to waive the requirements to obtain Informed Consent to collect these data on the care recipient is being made in accordance with 45 CFR 46. 116 and NIH OHSRP SOP 12 section 12.10: (A) An IRB may approve a consent procedure which does not include, or which alters, some or all of the elements of informed consent set forth in this section, or waive the requirements to obtain informed consent provided the IRB finds and documents that:

(1) The research involves no more than minimal risk to the subjects;

The clinical data requested includes broad categories such as disease type, and are information currently tracked in CRIS such as length of stay. The information involves no more than minimal risk for the HSCT patient (care recipient) and could be adequately covered under the general admission consent where patients are informed that “It [information in your medical record] is used for research by NIH scientist, some of whom have no personal contact with you”.

(2) The waiver or alteration will not adversely affect the rights and welfare of the subjects;

The requested waiver does not affect the rights of the HSCT patient (care recipient) as the data will not be shared with the caregiver subject and all identifiable information will be coded.

(3) The research could not practicably be carried out without the waiver or alteration; and

The HSCT patient (care recipient) information could not be reasonably obtained from the caregiver (research subject) as this information is generally poorly understood by the lay pubic (e.g. disease stage) and would affect the validity of the data collected. Although the patients theoretically could be consented, they are not the research subjects in this study and will have been verbally informed by the research team when approached to assess potential caregiver availability. The purpose of this information is to describe the caregiving experience (e.g. burden) not to describe the patient.

(4) Whenever appropriate, the subjects will be provided with additional pertinent information after participation.

The information collected from the patient’s record will only be used to characterize the caregiving experience. There will be no findings from the patient information to return to the patient although all study findings will be provided to the research subjects, the caregivers and the public.

Since the level of risk of this study is minimal risk, if the caregiver participant is not at the CC for initial enrollment, the consent will be sent to the participant to be executed over the telephone. The study will be explained to the caregiver including the objectives, time commitment and process. If the subject chooses to participate he/she will be directed to sign and date the consent and have someone witness and date his/her signature. The signed informed consent document will then be return-mailed to the principal or associate investigator who led the discussion, who will sign and date and mail back a fully executed copy for the subject’s records. The informed consent process will be documented in CRIS and/or a progress note. The original informed consent will be filed in the subject’s medical record and/or research record as appropriate. (***See Clinical Center Policy and Communication Bulletin M77-2 Dated 21 May 2011. Subject: Informed Consent, Section X. Consent from someone not at the clinical center: “For research protocols or any procedures performed for the purposes of research that involve obtaining consent via technology and/or electronic process, rather than in person, the procedures for obtaining consent, including how information will be transmitted and documented and by whom, shall be detailed in the written protocol. Review and approval must first be obtained from the institute clinical director and the relevant IRB).*

## 10.4 Research Subject Compensation

Enrolled subjects will be compensated for inconveniences based on guidelines for NIH Research Participant compensation. Research subjects will receive $15 for each blood sample and $40 for each outpatient visit (registration, H&P and/or clinic follow-ups). A study completion bonus of $50 will be offered for completing **all** study procedures including all clinic visits, laboratory samples and questionnaires completion, for a maximum compensation of $170 for participation. A maximum of no more than 550 mL of blood in an eight week period will be drawn.

An enrolled subject could therefore receive:

1^st^ visit $55 (registration, H&P, collection of blood, surveys)

2-Telephone follow-ups $10 (subjects in intervention group only)

2^nd^ visit $55 (collection of blood, clinic visit, surveys)

Bonus $50 (for completing **all** study procedures)

**Total potential study compensation: $160-170***

*depending on randomization

| **SEQUENCE FOR YOGA PRACTICE AUDIO FILE** | |
| --- | --- |
| **POSE OR PRACTICE** | **DESCRIPTION** |
| Seated Forward Bend | Sitting on a chair facing a desk, bed, or table that is higher than waist height), subjects lean forward and rest their arms and head on the table or desk. Subjects can cross arms and rest their head on crossed arms for cushion. Observe breath in and out. Remain in pose for 1-2 minutes. |
| Upward facing Hands | Subjects will stand facing wall (about 1 foot away from wall) with feet hip distance apart and hands on wall at shoulder height. On an exhalation, subjects will stretch arms straight by moving their hands slowly up the wall. If comfortable, subjects can rest forehead on the wall. Hold 15 seconds. |
| Downward facing Dog with Wall | Subjects stand facing wall with hands on wall. Subjects step back and bend at the hips until their arms, and legs are straight and their back is straight, like a table top. Stretch the arms and legs, and breath normally. Hold for 20 seconds. |
| Seated Arms Overhead Pose | Sitting in a stable chair with both feet on the floor, raise arms overhead and stretch them straight. Breath normally and hold for 15-20 seconds. |
| Chair Seated Twist | Sitting upright in chair with both feet on the floor, inhale and raise the left arm overhead. On an exhalation, keeping the legs and hips stable, twist at the waist to the right to take the hands to the right-hand arm or back of the chair. Stay twisting to the right and breath normally for 15-20 seconds |
| Seated Relaxation Pose | Place the chair with its back against the wall, and sit comfortably in the chair with both feet on the floor and the hands/arms supported on either the arms of the chair or the lap. Rest the back of the head against the chair or wall, if comfortable. Close the eyes and let go completely. Relax the feet and legs, the hips, the belly, the rib cage and chest, shoulders, arms, and fingertips. Relax the skin of the face, and feel as if you could soften the bones of the face (the lower jaw, the cheekbones, the bridge of the nose, the temples, and the bones of the forehead). Soften the inner corners of the eyes. Soften the tongue and let it rest in the floor of the mouth. Let go completely and observe the breath as it moves in and out (subjects are led through these instructions for approximately 5 minutes) |
| Seated Breath Work | Remaining in the same position as relaxation pose, subjects should begin to slowly deepen their inhalations. The breath should be long, slow, soft and very smooth, with no straining (1-2 minutes). Subjects will then resume normal breathing, then begin to slowly deepen their exhalations in the same manner (1-2 minutes), maintaining the relaxation of the body and the softness of the face, tongue, and eyes. |
| Seated Relaxation Pose | Allowing the breath to return to normal, subjects will repeat the guided relaxation pose above (approx. 3-5 minutes) |

Appendix A: Sequence for Yoga Practice Audio File

Appendix B: Treatment Fidelity

| **Focus Area**  **TREATMENT FIDELITY** | **Treatment Fidelity Indicators** |
| --- | --- |
| Design | - Standardized, audio file intervention - Intervention consistent with the theoretical framework - No yoga-based stress reduction intervention in the control group - Study designed to assess and control for non-treatment effects (medications, outside stress reduction techniques, outside life events/stressors |
| Training | - All investigators will be trained and have the manual of procedures to follow for all study activities and data management - Interventionist will receive training in delivering the audio file intervention in initial session from Advanced Iyengar Yoga Instructor including return demonstrations - Interventionist will train all study staff administering the initial intervention and will use return demonstration - Clinic staff interacting with study subjects and performing clinical assessments will be trained and the protocol to perform these assessments |
| Delivery | - Observation of delivery of random initial sessions by PI to ensure protocols are followed - Random observation of study staff of initial intervention sessions by certified Iyengar yoga instructor/interventionist to ensure content is appropriately delivered - Protocol deviations will be identified and corrected - Exit interview at end of study to address non-treatment effects - Data collection of non-treatment effects (medications, practice of stress reduction techniques, life events and stressors) |
| Receipt | - Documentation in the interventionist implementation log of ability of participant to perform poses and practices - Documentation in the interventionist implementation log of the subject’s ability to demonstrate the proper use of props (chair, wall, table) |
| Enactment | - Documentation in the interventionist implementation log of attendance at initial session - Practice diaries completed and collected - Phone calls every 2 weeks to assess practice and problems with practice and documented in the interventionist implementation log |

Appendix C: Demographic Interview


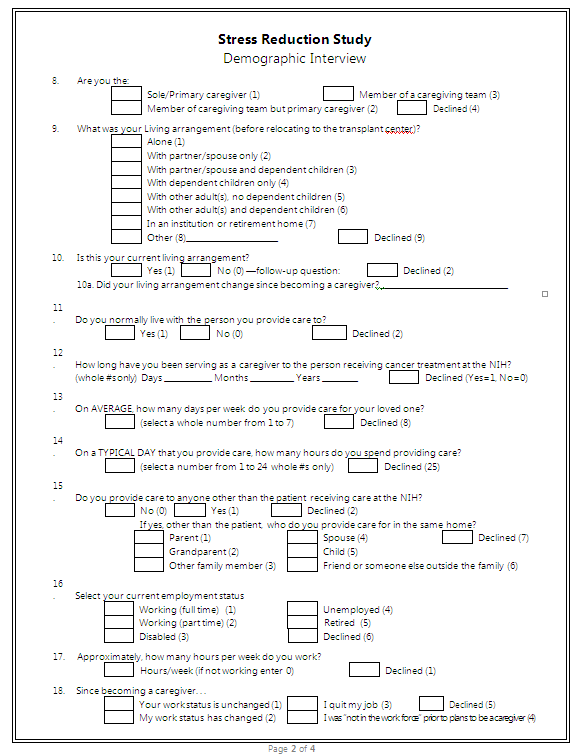


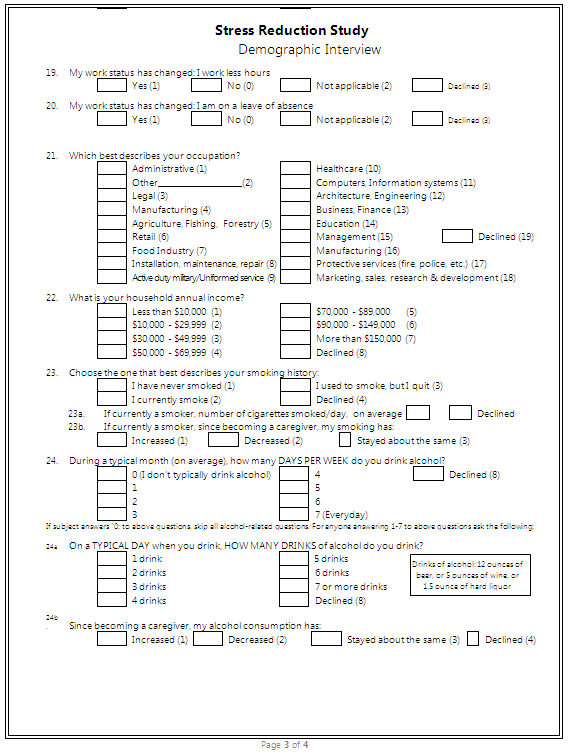

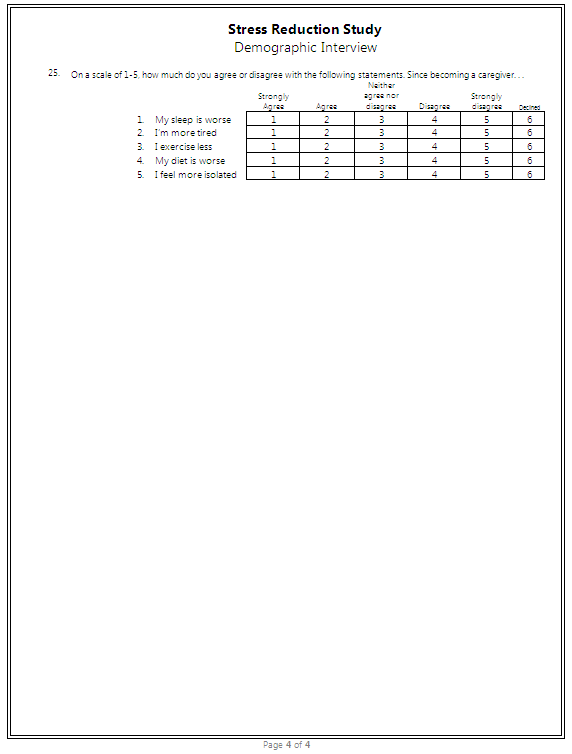
Appendix D: Telephone Script and Exit Interview

**Interviewer Instruction**: Intervention Group (Q1-7 for telephone follow-up at weeks 2 and 4, as well at exit interview at week 6); Control Group (Q8 at exit interview at week 6 ONLY)

[Thank the subjects for their participation in the study. Remind the subject of the study design and the plan to have a total of three calls, 2, 4 and 6 weeks]. Explain the purpose of the call: To evaluate frequency of practice –based on their diary (not memory) – and to identify barriers to daily practice. Confirm that they have their diary with them during the call.]

1. *How many times in the last two weeks have you practiced the complete audio (from beginning to end)?*
2. *On the days when you did not practice the entire audio CD, how many times did you start the audio but were unable to finish? [if >1, as them to ‘list’ the reasons for not completing]*
3. *On the days when you were not able to practice, please tell me what prevented you from practicing*
4. *Did you have any problems or issues when you were practicing?*
5. *In general, on a scale of 1 to 10, with one being not motivated and 10 being very motivated, how motivated were you to practice in the last 2 weeks?*
6. *In general, on a scale of 1 to 10, with one being not very confident and 10 being very confident, how confident are you that you will continue to practice?*
7. *Is there anything you would like to tell us about your experiences with your practice, or do you have any questions of us?*

**Exit Interview ONLY:**

1. *At the beginning of the study you received information on caregiver resources at the NIH Clinical Center (e.g. We Care Web site). Did you access that link or any other caregiver resources since the start of the study? If so, please describe.*

[Thank the subjects again for their participation in the study.]

**Appendix E: Subject Practice Log**

**11.0 References**

**1.** NAC. National Alliance for Caregiving. Caregiving in the U.S. 2009; <http://www.caregiving.org/data/Caregiving_in_the_US_2009_full_report.pdf>. Accessed 8/20/10.

**2.** Schulz R, Beach SR. Caregiving as a risk factor for mortality: the Caregiver Health Effects Study. *JAMA.* 1999;282(23):2215-2219. https://doi.org/10.1001/jama.282.23.2215

**3.** Schulz R, Sherwood PR. Physical and mental health effects of family caregiving. *The American journal of nursing.* 2008;108(9 Suppl). https://doi.org/10.1097/01.NAJ.0000336406.45248.4c

**4.** Christakis NA, Allison PD. Mortality after the hospitalization of a spouse. *N. Engl. J. Med.* 2006;354:719-730. https://doi.org/10.1056/NEJMsa050196

**5.** Capistrant BD, Moon JR, Berkman LF, Glymour MM. Current and long-term spousal caregiving and onset of cardiovascular disease. *J. Epidemiol. Community Health.* Oct 2012;66(10):951-956. https://doi.org/10.1136/jech-2011-200040

**6.** Lee S, Colditz GA, Berkman LF, Kawachi I. Caregiving and risk of coronary heart disease in U.S. women: A prospective study. *Am. J. Prev. Med.* 2003;24(2):113-119. <https://doi.org/10.1016/S0749-3797(02)00582-2>

**7.** Rohleder N, Marin TJ, Ma R, Miller GE. Biologic cost of caring for a cancer patient: Dysregulation of pro- and anti-inflammatory signaling pathways. *J. Clin. Oncol.* 2009;27(18):2909-2915. https://doi.10.1200/JCO.2008.18.7435

**8.** Mausbach BT, von Kanel R, Aschbacher K, et al. Spousal caregivers of patients with Alzheimer's disease show longitudinal increases in plasma level of tissue-type plasminogen activator antigen. *Psychosom. Med.* Oct 2007;69(8):816-822. https://doi.org/10.1097/PSY.0b013e318157d461

**9.** Miller GE, Murphy MLM, Cashman R, et al. Greater inflammatory activity and blunted glucocorticoid signaling in monocytes of chronically stressed caregivers. *Brain, Behavior, and Immunity.* 2014. https://doi.org/10.1016/j.bbi.2014.05.016

**10.** Fredman L, Cauley JA, Hochberg M, Ensrud KE, Doros G, Fractures SO. Mortality associated with caregiving, general stress, and caregiving-related stress in elderly women: Results of caregiver-study of osteoporotic fractures. *J. Am. Geriatr. Soc.* May 2010;58(5):937-943. https://doi.org/10.1111/j.1532-5415.2010.02808.x

**11.** Brown SL. Health effects of caregiving: Studies of helping behavior needed! *Alzheimer's Care Today.* 2007;8(3):235-246. https://doi: 10.1097/01.ALCAT.0000281871.81270.eb

**12.** Roth DL, Haley WE, Hovater M, Perkins M, Wadley VG, Judd S. Family caregiving and all-cause mortality: Findings from a population-based propensity-matched analysis. *Am. J. Epidemiol.* 2013;178(10):1571-1578. https://doi.org/10.1093/aje/kwt225

**13.** Beattie S, Lebel S. The experience of caregivers of hematological cancer patients undergoing a hematopoietic stem cell transplant: a comprehensive literature review. *Psycho-Oncol.* 2011;20(11):1137-1150. <https://doi.org/10.1002/pon.1962>

**14.** Keogh F, O'Riordan J, McNamara C, Duggan C, McCann SR. Psychosocial adaptation of patients and families following bone marrow transplantation: A prospective, longitudinal study. *Bone Marrow Transplant.* 1998;22(9):905-911. https://doi.org/10.1038/sj.bmt.1701443

**15.** Langer SL, Yi JC, Storer BE, Syrjala KL. Marital adjustment, satisfaction and dissolution among hematopoietic stem cell transplant patients and spouses: A prospective, five-year longitudinal investigation. *Psycho-Oncol.* 2010;19(2):190-200. https://doi.org/10.1002/pon.1542

**16.** Siston AK, List MA, Daugherty CK, et al. Psychosocial adjustment of patients and caregivers prior to allogeneic bone marrow transplantation. *Bone Marrow Transplant.* 2001;27(11):1181-1188. https://doi.org/10.1038/sj.bmt.1703059

**17.** Wright AA, Keating NL, Balboni TA, Matulonis UA, Block SD, Prigerson HG. Place of death: Correlations with quality of life of patients with cancer and predictors of bereaved caregivers' mental health. *J. Clin. Oncol.* 2010;28(29):4457-4464. https://doi.org/10.1200/JCO.2009.26.3863

**18.** Anderson WG, Arnold RM, Angus DC, Bryce CL. Posttraumatic stress and complicated grief in family members of patients in the intensive care unit. *J. Gen. Intern. Med.* Nov 2008;23(11):1871-1876. https://doi.org/10.1007/s11606-008-0770-2

**19.** Azoulay E, Pochard F, Kentish-Barnes N, et al. Risk of post-traumatic stress symptoms in family members of intensive care unit patients. *Am. J. Respir. Crit. Care Med.* May 1 2005;171(9):987-994. https://doi.org/10.1164/rccm.200409-1295OC

**20.** Northouse L, Williams AL, Given B, McCorkle R. Psychosocial care for family caregivers of patients with cancer. *J. Clin. Oncol.* 2012;30(11):1227-1234. https://doi.org/10.1200/JCO.2011.39.5798

**21.** Applebaum AJ, Breitbart W. Care for the cancer caregiver: A systematic review. *Palliat. Support. Care.* Oct 10 2012:1-22. https://doi.org/10.1017/S1478951512000594

**22.** Williams LA. Whatever it takes: Informal caregiving dynamics in blood and marrow transplantation. *Oncol. Nurs. Forum.* 2007;34(2):379-387. https://doi.org/10.1188/07.ONF.379-387

**23.** Rodakowski J, Skidmore ER, Rogers JC, Schulz R. Role of social support in predicting caregiver burden. *Arch. Phys. Med. Rehabil.* 2012;93(12):2229-2236. https://doi.org/10.1016/j.apmr.2012.07.004

**24.** Park EO, Schumacher KL. The state of the science of family caregiver-care receiver mutuality: A systematic review. *Nurs. Inq.* 2013;26(10):12032. https://doi.org/10.1111/nin.12032

**25.** Stenberg U, Ruland CM, Miaskowski C. Review of the literature on the effects of caring for a patient with cancer. *Psycho-Oncol.* 2010;19(10):1013-1025. https://doi.org/10.1002/pon.1670

**26.** Kotronoulas G, Wengström Y, Kearney N. Sleep patterns and sleep-impairing factors of persons providing informal care for people with cancer: A critical review of the literature. *Cancer Nurs.* 2013;36(1):E1-E15. https://doi.org/10.1097/NCC.0b013e3182456c38

**27.** Ross A, Yang L, Wehrlen L, Klagholz S, Bevans M. Do healthy behaviors have a relationship with sleep disturbances and fatigue in transplant caregivers? *Society of Behavioral Medicine*. Philadelphia, PA 2014.

**28.** Pollock EA, Litzelman K, Wisk LE, Witt WP. Correlates of physiological and psychological stress among parents of childhood cancer and brain tumor survivors. *Academic Pediatrics.* 2013;13(2):105-112. https://doi.org/10.1016/j.acap.2012.11.005

**29.** Swore Fletcher B, Dodd M. Symptom experience of family caregivers of patients with cancer. *Oncol. Nurs. Forum.* 2008;35(2):E23-E44. https://doi.org/10.1188/08.ONF.E23-E44

**30.** Cho MH, Dodd MJ, Lee KA, Padilla G, Slaughter R. Self-reported sleep quality in family caregivers of gastric cancer patients who are receiving chemotherapy in Korea. *Journal of cancer education : the official journal of the American Association for Cancer Education.* 2006;21(1 Suppl):S37-41. https://doi.org/10.1207/s15430154jce2101s_8

**31.** Ross A, Sundaramurthi T, Bevans M. A labor of love: The influence of cancer caregiving on health behaviors. *Cancer Nurs.* 2012;36(6), 474-483.https://doi.org/10.1097/NCC.0b013e3182747b75

**32.** Beesley VL, Price MA, Webb PM. Loss of lifestyle: Health behaviour and weight changes after becoming a caregiver of a family member diagnosed with ovarian cancer. *Support. Care Cancer.* 2011;19(12):1949-1956. https://doi.org/10.1007/s00520-010-1035-2

**33.** Hoffman GJ, Lee J, Mendez-Luck CA. Health behaviors among Baby Boomer informal caregivers. *Gerontologist.* 2012;52(2):219-230. https://doi.org/10.1093/geront/gns003

**34.** Lee CJ. A comparison of health promotion behaviors in rural and urban community-dwelling spousal caregivers. *J Gerontol Nurs.* 2009;35(5):34-40. https://doi.org/10.3928/00989134-20090331-04

**35.** McElligott D, Capitulo KL, Morris DL, Click ER. The effect of a holistic program on health-promoting behaviors in hospital registered nurses. *Journal of holistic nursing : official journal of the American Holistic Nurses' Association.* 2010;28(3):175-183. https://doi.org/10.1177/0898010110368860

**36.** Bevans MF, Castro K, Wehrlen L, et al. Healthy lifestyles may improve transplant caregiver outcomes. *Biology of blood and marrow transplantation : journal of the American Society for Blood and Marrow Transplantation.* 2010;16(2):S193.

**37.** Hurley RV, Patterson TG, Cooley SJ. Meditation-based interventions for family caregivers of people with dementia: a review of the empirical literature. *Aging Ment. Health.* 2014;18(3):281-288. https://doi.org/10.1080/13607863.2013.837145

**38.** Epstein-Lubow G, McBee L, Darling E, Armey M, Miller IW. A Pilot investigation of mindfulness-based stress reduction for caregivers of frail elderly. *Mindfulness.* 2011;2(2):95-102. [https://doi.org/10.1007/s12671-011-0047-4](https://psycnet.apa.org/doi/10.1007/s12671-011-0047-4)

**39.** Hou RJ, Wong SYS, Yip BHK, et al. The effects of mindfulness-based stress reduction program on the mental health of family caregivers: A randomized controlled trial. *Psychother. Psychosom.* 2013;83(1):45-53. https://doi.org/10.1159/000353278

**40.** Whitebird RR, Kreitzer M, Lauren Crain A, Lewis BA, Hanson LR, Enstad CJ. Mindfulness-based stress reduction for family caregivers: A randomized controlled trial. *Gerontologist.* 2013;53(4):676-686. https://doi.org/10.1093/geront/gns126

**41.** Kogler M, Brandstatter M, Borasio GD, Fensterer V, Kuchenhoff H, Fegg MJ. Mindfulness in informal caregivers of palliative patients. *Palliat. Support. Care.* 2013:13(1);11-18. https://doi.org/10.1017/S1478951513000400

**42.** Danucalov MAD, Kozasa EH, Ribas KT, et al. A Yoga and compassion meditation program reduces stress in familial caregivers of alzheimer's disease patients. *Evid. Based Complement. Alternat. Med.* 2013;2013. <https://doi.org/10.1155/2013/513149>

**43.** Lavretsky H, Epel ES, Siddarth P, et al. A pilot study of yogic meditation for family dementia caregivers with depressive symptoms: effects on mental health, cognition, and telomerase activity. *Int. J. Geriatr. Psychiatry.* 2013;28(1):57-65.

**44.** Northouse LL, Katapodi MC, Song L, Zhang L, Mood DW. Interventions with family caregivers of cancer patients, Meta-analysis of randomized trials. *CA Cancer Journal for Clinicians.* 2010;60(5):317-339. https://doi.org/10.1002/gps.3790

**45.** Lengacher CA, Kip KE, Barta M, et al. A pilot study evaluating the effect of mindfulness-based stress reduction on psychological status, physical status, salivary cortisol, and interleukin-6 among advanced-stage cancer patients and their caregivers. *J. Holist. Nurs.* Sep 2012;30(3):170-185. https://doi.org/10.1177/0898010111435949

**46.** Keir ST. Levels of stress and intervention preferences of caregivers of brain tumor patients. *Cancer Nurs.* 2007;30(6):E33-E39. https://doi.org/10.1097/01.NCC.0000300174.18584.f9

**47.** Bevans M. 11-CC-0265 - A pilot study to examine physiological and clinical markers of chronic stress in caregivers of allogeneic hematopoietic stem cell transplant (HSCT) recipients. 2011.

**48.** Grundy SM, Brewer HB, Cleeman JI, Smith SC, Lenfant C, Participants C. Definition of metabolic syndrome - Report of the National Heart, Lung, and Blood Institute/American Heart Association Conference on Scientific Issues Related to Definition. *Circulation.* Jan 27 2004;109(3):433-438. https://doi.org/10.1161/01.CIR.0000111245.75752.C6

**49.** Third Report of the National Cholesterol Education Program (NCEP) Expert Panel on Detection, Evaluation, and Treatment of High Blood Cholesterol in Adults (Adult Treatment Panel III) final report. *Circulation.* 2002;106(25):3143-3421.

**50.** Sniderman AD, Marcovina SM. Apolipoprotein A1 and B. *Clin. Lab. Med.* Dec 2006;26(4):733-750.

**51.** Yusuf S, Hawken S, Ounpuu S, et al. Effect of potentially modifiable risk factors associated with myocardial infarction in 52 countries (the INTERHEART study): case-control study. *Lancet.* 2004;364(9438):937-952. https://doi.org/10.1016/S0140-6736(04)17018-9

**52.** Vitaliano PP, Murphy M, Young HM, Echeverria D, Borson S. Does caring for a spouse with dementia promote cognitive decline? A hypothesis and proposed mechanisms. *J. Am. Geriatr. Soc.* 2011;59(5):900-908. https://doi.org/10.1111/j.1532-5415.2011.03368.x

**53.** Sharma VK, M R, S V, et al. Effect of fast and slow pranayama practice on cognitive functions in healthy volunteers. *Journal of clinical and diagnostic research : JCDR.* Jan 2014;8(1):10-13. https://doi.org/10.7860/JCDR/2014/7256.3668

**54.** Bonura KB, Tenenbaum G. Effects of yoga on psychological health in older adults. *J. Phys. Act. Health.* 2014;11(7):1334–1341.. https://doi.org/10.1123/jpah.2012-0365

**55.** Park J, McCaffrey R, Newman D, Cheung C, Hagen D. The effect of sit 'n' fit chair yoga among community-dwelling older adults with osteoarthritis. *Holist. Nurs. Pract.* 2014;28(4):247-257. https://doi.org/10.1097/HNP.0000000000000034

**56.** Given CW, Given B, Stommel M, Collins C, King S, Franklin S. The caregiver reaction assessment (CRA) for caregivers to persons with chronic physical and mental impairments. *Res. Nurs. Health.* 1992;15(4):271-283.

**57.** Locke BZ, Putnam P. Center for Epidemiologic Studies Depression Scale (CES-D SCALE). 1977; <http://www.friendsnrc.org/download/outcomeresources/toolkit/annot/cesd.pdf>. Accessed 07/25/2008.

**58.** Katz S, Ford A, Moskowitz R. Studies of illness in the aged. The index of ADL: A standardized measure of biological and psychosocial function. *JAMA.* 1963;185:221-229.

**59.** Salsman JM, Lai JS, Hendrie HC, et al. Assessing psychological well-being: self-report instruments for the NIH Toolbox. *Qual. Life Res.* 2014;23(1): 205–215. https://doi.org/10.1007/s11136-013-0452-3

**60.** Noble Walker S, Richert Sechrist K, Pender NJ. The Health-Promoting Lifestyle Profile: Development and psychometric characteristics. *Nurs. Res.* 1987;36(2):76-80.

**61.** Walker SN, Hill-Polerecky DM. Psychometric evaluation of the Health-Promoting Lifestyle Profile II 1996.

**62.** Callaghan DM. Health-promoting self-care behaviors, self-care self-efficacy, and self-care agency. *Nurs. Sci. Q.* 2003;16(3):247-254. https://doi.org/10.1177/0894318403016003016

**63.** Cella D, Riley W, Stone A, et al. The Patient-Reported Outcomes Measurement Information System (PROMIS) developed and tested its first wave of adult self-reported health outcome item banks: 2005-2008. *J. Clin. Epidemiol.* Nov 2010;63(11):1179-1194. https://doi.org/10.1016/j.jclinepi.2010.04.011

**64.** Dolce MC. The nternet as a Source of Health Information: Experiences of Cancer Survivors and Caregivers With Healthcare Providers. *Oncol. Nurs. Forum.* May 2011;38(3):353-359.

**65.** Baker F. *Item Response Theory: Parameter Estimation Techniques*: Marcel Dekker; 2004.

**66.** Buysse DJ, Reynolds CF, Monk TH, Berman SR, Kupfer DJ. The Pittsburgh sleep quality index: A new instrument for psychiatric practice and research. *Psychiatry Res.* 1989;28(2):193-213.

**67.** Beck SL, Schwartz AL, Towsley G, Dudley W, Barsevick A. Psychometric evaluation of the Pittsburgh sleep quality index in cancer patients. *J. Pain Symptom Manage.* 2004;27(2):140-148. https://doi.org/10.1016/j.jpainsymman.2003.12.002

**68.** Berger AM, Parker KP, Young-McCaughan S, et al. Sleep wake disturbances in people with cancer and their caregivers: state of the science. *Oncol. Nurs. Forum.* 2005;32(6):E98-126. https://doi.org/10.1188/05.ONF.E98-E126

**69.** Carlson LE, Garland SN. Impact of mindfulness-based stress reduction (MBSR) on sleep, mood, stress and fatigue symptoms in cancer outpatients. *International Journal of Behavioral Medicine.* 2005;12(4):278-285. https://doi.org/10.1207/s15327558ijbm1204_9

**70.** Carpenter JS, Andrykowski MA. Psychometric evaluation of the Pittsburgh Sleep Quality Index. *J. Psychosom. Res.* 1998;45(1):5-13.

**71.** Cohen L, Warneke C, Fouladi RT, Rodriguez MA, Chaoul-Reich A. Psychological adjustment and sleep quality in a randomized trial of the effects of a Tibetan yoga intervention in patients with lymphoma. *Cancer.* 2004;100(10):2253-2260. https://doi.org/10.1002/cncr.20236

**72.** Fortner BV, Stepanski EJ, Wang SC, Kasprowicz S, Durrence HH. Sleep and quality of life in breast cancer patients. *J. Pain Symptom Manage.* 2002;24(5):471-480. https://doi.org/10.1016/s0885-3924(02)00500-6

**73.** Gross CR, Kreitzer MJ, Russas V, Treesak C, Frazier PA, Hertz MI. Mindfulness meditation to reduce symptoms after organ transplant: A pilot study. *Adv. Mind Body Med.* 2004;20(2):20-29.

**74.** Le Guen Y, Gagnadoux F, Hureaux J, et al. Sleep disturbances and impaired daytime functioning in outpatients with newly diagnosed lung cancer. *Lung Cancer.* 2007;58(1):139-143. https://doi.org/10.1016/j.lungcan.2007.05.021

**75.** Mystakidou K, Parpa E, Tsilika E, et al. Sleep quality in advanced cancer patients. *J. Psychosom. Res.* 2007;62(5):527-533. https://doi.org/10.1016/j.jpsychores.2006.11.008

**76.** Owen DC, Parker KP, McGuire DB. Comparison of subjective sleep quality in patients with cancer and healthy subjects. *Oncol. Nurs. Forum.* 1999;26(10):1649-1651.

**77.** Simeit R, Deck R, Conta-Marx B. Sleep management training for cancer patients with insomia. *Support. Care Cancer.* 2004;12(3):176-183. https://doi.org/10.1007/s00520-004-0594-5

**78.** Vena C, Parker KP, Allen R, Bliwise DL, Jain S, Kimble L. Sleep-wake disturbances and quality of life in patients with advanced lung cancer. *Oncol. Nurs. Forum.* 2006;33(4):761-769. https://doi.org/10.1188/06.ONF.761-769

**79.** Walach H, Buchheld N, Buttenmuller V, Kleinknecht N, Schmidt S. Measuring mindfulness - the Freiburg Mindfulness Inventory (FMI). *Personality and Individual Differences.* 2006;40(8):1543-1555. <https://doi.org/10.1016/j.paid.2005.11.025>

**80.** Stein KD, Jacobsen PB, Blanchard CM, Thors C. Further validation of the multidimensional fatigue symptom inventory-short form. *J. Pain Symptom Manage.* 2004;27(1):14-23. https://doi.org/10.1016/j.jpainsymman.2003.06.003

**81.** Schwartz AH. Validity of cancer-related fatigue instruments. *Pharmacotherapy.* 2002;22(11):1433-1441. https://doi.org/10.1592/phco.22.16.1433.33690

**82.** Lim W, Hong S, Nelesen R, Dimsdale JE. The association of obesity, cytokine levels, and depressive symptoms with diverse measures of fatigue in healthy subjects. *Arch. Intern. Med.* 2005;165(8):910-915. https://doi.org/10.1001/archinte.165.8.910

**83.** De Leeuw R, Studts JL, Carlson CR. Fatigue and fatigue-related symptoms in an orofacial pain population. *Oral Surg. Oral Med. Oral Pathol. Oral Radiol. Endod.* 2005;99(2):168-174. https://doi.org/10.1016/j.tripleo.2004.03.001

**84.** Charlson M. A new method of classifying prognostic comorbidity in longitudinal studies: Development and validation. *J. Chronic Dis.* 1987;40(5):373-383. https://doi.org/10.1016/0021-9681(87)90171-8

**85.** Cavanagh K, Strauss C, Cicconi F, Griffiths N, Wyper A, Jones F. A randomised controlled trial of a brief online mindfulness-based intervention. *Behav. Res. Ther.* 2013;51(9):573-578. https://doi.org/10.1016/j.brat.2013.06.003

**86.** Fitzmaurice GM, Laird NM, Ware JH. *Applied Longitudinal Analysis*: Wiley; 2011.

**87.** Norman GR, Sloan JA, Wyrwich KW. Interpretation of changes in health-related quality of life - The remarkable universality of half a standard deviation. *Med. Care.* 2003;41(5):582-592. https://doi.org/10.1097/01.MLR.0000062554.74615.4C
